# SDS3 regulates microglial inflammation by modulating the expression of the upstream kinase ASK1 in the p38 MAPK signaling pathway

**DOI:** 10.1007/s00011-024-01913-5

**Published:** 2024-07-15

**Authors:** Jian Shen, Wenjia Lai, Zeyang Li, Wenyuan Zhu, Xue Bai, Zihao Yang, Qingsong Wang, Jianguo Ji

**Affiliations:** 1 Department of General Surgery, Beijing Chao-Yang Hospital, Capital Medical University, Beijing, 100020 China; 2 State Key Laboratory of Protein and Plant Gene Research, College of Life Sciences, Peking University, Beijing, 100871 China; 3 Division of Nanotechnology Development, National Center for Nanoscience and Technology, Beijing, 100190 China

**Keywords:** Microglia, Neuroinflammation, SDS3, p38 MAPK, ASK1

## Abstract

**Background:**

Microglia, the main innate immune cells in the central nervous system, are key drivers of neuroinflammation, which plays a crucial role in the pathogenesis of neurodegenerative diseases. The Sin3/histone deacetylase (HDAC) complex, a highly conserved multiprotein co-repressor complex, primarily performs transcriptional repression via deacetylase activity; however, the function of SDS3, which maintains the integrity of the complex, in microglia remains unclear.

**Methods:**

To uncover the regulatory role of the transcriptional co-repressor SDS3 in microglial inflammation, we used chromatin immunoprecipitation to identify SDS3 target genes and combined with transcriptomics and proteomics analysis to explore expression changes in cells following SDS3 knocking down. Subsequently, we validated our findings through experimental assays.

**Results:**

Our analysis revealed that SDS3 modulates the expression of the upstream kinase ASK1 of the p38 MAPK pathway, thus regulating the activation of signaling pathways and ultimately influencing inflammation.

**Conclusions:**

Our findings provide important evidence of the contributions of SDS3 toward microglial inflammation and offer new insights into the regulatory mechanisms of microglial inflammatory responses.

**Supplementary Information:**

The online version contains supplementary material available at 10.1007/s00011-024-01913-5.

## Introduction

Neurodegenerative diseases (NDs) are characterized by progressive neuron loss, resulting in severe motor impairments, cognitive decline, and dementia. The global incidence of NDs exceeds 40 million individuals and is strongly associated with advanced age [1]. The most common NDs include Alzheimer’s disease (AD), Parkinson’s disease (PD), Huntington’s disease, and amyotrophic lateral sclerosis. Shared pathological features include neuronal dysfunction, aberrant protein aggregation, oxidative stress, programmed cell death, and neuroinflammation [2]. The molecular mechanisms underlying ND pathogenesis remain unclear, limiting therapeutic options for symptomatic relief. Consequently, understanding these mechanisms is crucial for the development of effective treatments.

Neuroinflammation is the central nervous system’s response to homeostatic imbalance and involves numerous cell types, such as microglia, astrocytes, and oligodendrocytes, the blood–brain barrier, cytokines, and cytokine signaling pathways [3]. Depending on the specific context triggering the inflammatory response, neuroinflammation can have either beneficial or detrimental effects. AD and PD are characterized by microglial and astrocytic activation and elevated levels of inflammatory mediators [4]. Genetic studies have also highlighted the association between inflammation-regulating genes and NDs [5–10], emphasizing the important role of neuroinflammation in disease pathogenesis.

Microglia, as the primary innate immune cells in the central nervous system, are essential for brain development, the maintenance of homeostasis, and response to infections. Under pathological conditions associated with aging and NDs, microglia undergo excessive activation and functional dysregulation, resulting in impaired degradation, heightened inflammatory responses, production of proinflammatory cytokines [11, 12], generation of reactive oxygen species [13, 14], and neurotoxicity, thereby exacerbating ND pathologies [15, 16]. Investigating the regulatory mechanisms of microglial inflammatory responses is crucial for a deeper understanding of ND pathogenesis.

Histone deacetylases (HDACs) are enzymes that catalyze the removal of acetyl groups from acetylated lysine residues on histone and nonhistone proteins. HDAC1/2 can form four classical transcriptional co-repressor complexes, and the Sin3/HDAC complex is one of the classical multiprotein complexes formed by HDAC1/2 [17]. In mammalian cells, the core components of the Sin3/HDAC complex include paired amphipathic helix protein Sin3A/B (Sin3A/B), HDAC1/2, the Sin3 histone deacetylase corepressor complex component SDS3 (SDS3), retinoblastoma-binding protein 4/7 (RBBP4/7), 30-kDa Sin3-associated polypeptide (SAP30), and 18-kDa Sin3-associated polypeptide (SAP18). The Sin3/HDAC complex plays a critical role in processes such as the cell cycle, cell proliferation, and cellular senescence [18, 19]. The classical view is that Sin3/HDAC represses transcription through histone deacetylase activity and is therefore referred to as a transcriptional co-repressor complex [20, 21].

Some existing studies have revealed the regulatory role of Sin3/HDAC in inflammatory responses. In ovarian clear cell carcinoma cells with *PIK3CA* mutations, knockdown of *ARID1A* impedes recruitment of the Sin3A/HDAC complex to the promoters of cytokines genes, such as *IL6* and *IL8*, releasing transcriptional repression and promoting cytokine production and cancer progression [22]. In mouse macrophages, lipopolysaccharide (LPS) treatment induces recruitment of the Sin3A/HDAC complex to the promoter region of the gene encoding inducible nitric oxide synthase (iNOS), thereby inhibiting iNOS expression [23]. In human macrophages, the Sin3A/HDAC complex can bind to the promoter regions of interferon (IFN) response genes, such as *IFNB1* and *IRF7*, as well as proinflammatory genes, such as *TNFS* and *CCL3*. After LPS treatment, the binding of Sin3A to these promoter regions is reduced, resulting in upregulated gene expression [24]. Together, these studies suggest that the Sin3/HDAC complex negatively regulates inflammatory responses through transcriptional repression in different contexts.

SDS3 is one of the core components of the Sin3/HDAC complex and plays a critical role in maintaining the integrity and histone deacetylase activity of the complex [25, 26]. However, current research on the biological functions of SDS3 in regulating the Sin3/HDAC complex is limited and mainly focuses on cell growth and tissue development [27]. There are few reports on the function of SDS3 in regulating inflammatory responses in microglia.

Here, we examine changes in the expression of SDS3 in LPS-stimulated microglia and investigate the impact of SDS3 on microglial inflammatory responses. Using tandem mass tags (TMT)-labeled quantitative proteomics and RNA-sequencing (RNA-seq) transcriptomics techniques, we analyze the effects of SDS3 on gene and protein expression in microglia and characterize its potential biological functions. Furthermore, by using chromatin immunoprecipitation with sequencing (ChIP-seq) technology and integrating proteomics and transcriptomics data, we identify downstream genes regulated by SDS3 in microglia. Together, we reveal the molecular mechanisms through which SDS3 regulates microglial inflammatory responses, thereby offering new insights into the biological function of SDS3 and the regulatory mechanisms of microglial inflammation.

## Materials and methods

### Cells and plasmids

BV2 mouse microglial cells, primary mouse microglia, and SV40T-transformed human embryonic kidney 293T (HEK 293T) cells were obtained from the Cell Resource Center, iCell Bioscience, Inc. (MIC-iCell-n010), and Peking Union Medical College. The lentiCRISPR v2 plasmid; pMDLg/pRRE, pRSV-Rev, and pMD2.G plasmids; and pBluescript II KS(-) plasmid were kindly provided by the laboratories of Professor Yi Rao, Professor Chen Zhang, and Professor Daochun Kong, respectively (all at Peking University).

The detailed procedures and parameters of the experiments are described in the Supplementary information.

### Cell culture

BV2 mouse microglial cells, primary mouse microglia, and HEK 293T cells were cultured in high-glucose DMEM (Hyclone, SH30022.01) supplemented with 10% fetal bovine serum (Hyclone, SV30087.03) in a humidified incubator at 37 °C with 5% CO_2_. When cells reached 80–100% confluency, they were dissociated with 0.25% trypsin solution (Hyclone, SH30042.01) and passaged into new culture dishes as needed.

### Small interfering RNA (siRNA) transfection

Control siRNA (sc-37,007), SDS3 siRNA (sc-153,291), and HDAC1 siRNA (sc-29,344) were obtained from Santa Cruz and were transfected into BV2 cells using Lipofectamine RNAiMAX (Invitrogen, 13,778,030). Six to 8 h after transfection, the culture medium was replaced, and subsequent experiments were conducted 24 h later.

### CRISPR–Cas9

Using the Optimized CRISPR Design tool, sgRNAs were designed for *ASK1*-knockout and control cells. Oligonucleotides were ligated into the lentiCRISPR v2 plasmid, transformed into TransStbl3 *Escherichia coli* cells, and cultured on ampicillin LB agar. Plasmids were then extracted and sequenced. The lentiCRISPR v2 plasmid containing sgRNA and the pMDLg/pRRE, pRSV-Rev, and pMD2.G plasmids were transfected into HEK 293T cells using polyethylenimine to package the lentivirus. Viral suspensions were added to BV2 cells, and puromycin selection was performed. Genomic DNA from selected clonal cells was extracted, and the *ASK1* locus was amplified by PCR, ligated into the pBluescript II KS(-) plasmid, transformed into Trans5α *Escherichia coli* cells, and cultured on ampicillin LB agar. Colonies were sequenced for locus verification.

### LPS treatment

After transfection with SDS3 siRNA or Control siRNA, BV2 cells were treated with 1 μg/mL LPS for 1 h. After treatment, the cells were collected to assess the activation of the p38 MAPK pathway. Additionally, the transfected cells were treated with 1 μg/mL LPS for 6 h, and the protein and mRNA expression levels of inflammatory factors were measured. For the detection of nitric oxide, transfected cells were treated with 1 μg/mL LPS for 24 h before collection. As a comparison, BV2 cells were treated with 20 μM p38 MAPK inhibitor (SB203580) or DMSO for 1 h, followed by treatment with 1 μg/mL LPS for 6 h and subsequent Western blotting and real-time quantitative PCR (qPCR).

After treating *ASK1*-knockout BV2 cells and control BV2 cells with 1 μg/mL LPS for 1 h, the cells were collected to assess the activation state of the p38 MAPK pathway. Transfected cells were treated with 1 μg/mL LPS for 6 h, and the protein levels of inflammatory factors were measured. For the detection of nitric oxide, the transfected cells were treated with 1 μg/mL LPS for 24 h before sample collection.

After transfecting *ASK1*-knockout BV2 cells and control BV2 cells with SDS3 siRNA or Control siRNA, the cells were treated with 1 μg/mL LPS for 6 h, followed by Western blotting and qPCR to determine the expression of inflammatory factors.

### Real-time qPCR and Western blotting

After transfecting BV2 cells with siRNA and treating with LPS, total RNA was extracted using an EasyPure RNA kit (Transgenbiotech, ER101). *ACTB* was used as the reference gene, and primer sequences are provided in Supplemental Table S1.

To obtain protein used for Western blotting, after treatment, BV2 cells and primary mouse microglia were lysed using a 1% SDS lysis buffer and sonicated to disrupt nucleic acids. Primary antibodies for Western blotting analysis are provided in Supplemental Table S2. β-Actin served as the loading control.

### Nitric oxide measurement

Assessment of nitric oxide levels was performed using a nitric oxide assay kit (Beyotime, S0021). The nitrite concentration in the cell culture supernatant was measured by spectrophotometry to evaluate the nitric oxide levels.

### Proteomic analysis

BV2 cells were transfected with Control siRNA and SDS3 siRNA in six-well plates. Cells were collected and lysed as previously described [28], and peptides were labeled with a TMT 6-plex Isobaric Label Reagent set (Thermo Scientific, 90,064) according to the manufacturer’s instructions. Labeled peptides were then purified using a C18 solid-phase extraction column (Empore) for desalting and then freeze-dried. Peptides were analyzed by Orbitrap Fusion Lumos Tribrid mass spectrometer (Thermo Scientific).

### RNA-seq

BV2 cells were transfected with Control siRNA and SDS3 siRNA in six-well plates. Following incubation, the culture medium was aspirated, and 1 mL of TRIzol Reagent (Invitrogen, 15,596,026) was added to lyse the cells. Cell lysates were transferred to 2-mL EP tubes and flash-frozen in liquid nitrogen. Subsequent processing was performed by Suzhou Geneweave Biotechnology Co., Ltd. Differential gene expression analysis was performed using the DESeq2 package from Bioconductor in R [29].

### Bioinformatics analysis and statistical methods

Gene Ontology (GO) enrichment analysis of differentially expressed proteins and genes was performed using the ClueGO plugin in Cytoscape [30]. All experiments were conducted with biological and technical replicates, as specified. Statistical significance was defined as indicated and specified in the figure and table captions. If not otherwise mentioned, statistical significance was evaluated using GraphPad Prism 7 software (version 7.00) with a one-way analysis of variance (ANOVA), and a *p* value of < 0.05 was considered statistically significant. Data are presented as mean ± standard error of the mean (SEM) from at least three independent experiments.

### ChIP-seq and ChIP-qPCR

BV2 cells were cultured in 15-cm dishes and cross-linked with 1% formaldehyde. ChIP experiments were performed using a SimpleChIP Enzymatic Chromatin IP kit (magnetic beads; Cell Signaling Technology, 9003) according to the manufacturer’s instructions. DNA that was enriched following ChIP with anti-SDS3 (Supplemental Table S2) and input DNA were sent to Suzhou Geneweave Biotechnology Co., Ltd., for further processing.

DNA enriched after ChIP with anti-SDS3, anti-HDAC1, and anti-control (Supplemental Table S2) was subjected to fluorescence-based qPCR. Three primer sets targeting the *ASK1* promoter region were designed (Supplemental Table S1), and the relative amount of *ASK1* promoter region DNA in each sample compared with that in the input sample was calculated using the ΔΔC_*t*_ method based on the *C*_*t*_ values. Results are presented as a percentage of input.

## Results

### LPS-stimulated microglia exhibit decreased SDS3 expression

**Fig. 1:**
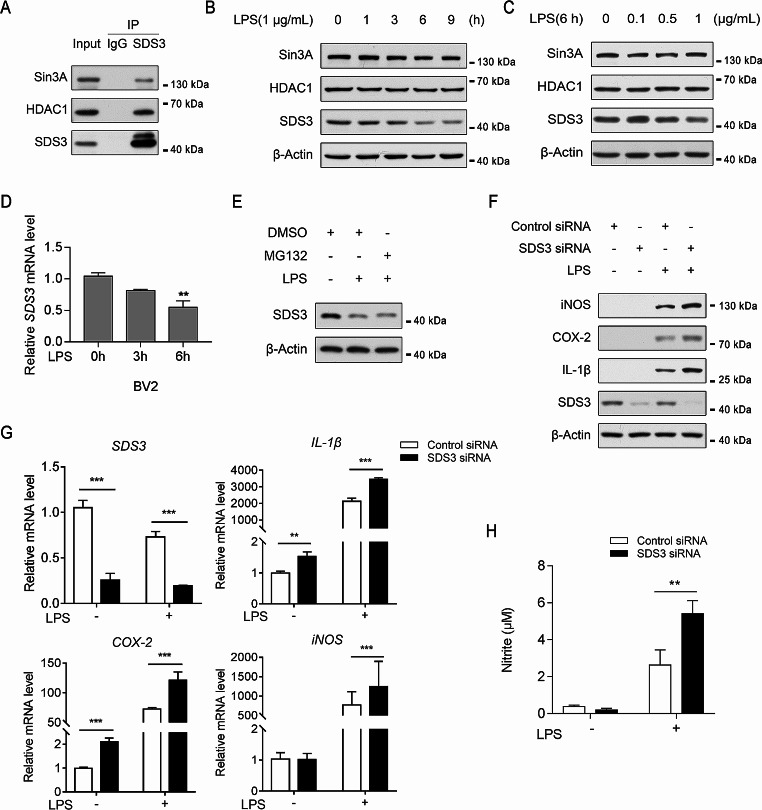
Knockdown of SDS3 enhances lipopolysaccharide (LPS)-induced inflammatory responses in microglia. (**A**) Immunoprecipitation (IP) followed by Western blotting confirmed the interaction between SDS3, Sin3A, and HDAC1 in BV2 cells. (**B**) Protein levels of SDS3, Sin3A, and HDAC1 in BV2 cells after treatment with 1 μg/mL LPS for different lengths of time. β-Actin was used as the loading control. (**C**) Protein levels of SDS3, Sin3A, and HDAC1 in BV2 cells after treatment with different concentrations of LPS for 6 h. β-Actin was used as the loading control. (**D**) Changes in SDS3 mRNA expression levels in BV2 cells after treatment with 1 μg/mL LPS for different lengths of time. *ACTB* was used as the reference gene. The control group represents 0 h; *n* = 3; error bars represent mean ± standard error of the mean (SEM). Data were analyzed by one-way analysis of variance; *p < 0.05. (**E**) Protein levels of SDS3 in BV2 cells after treatment with 4 μM MG132 or DMSO for 1 h, followed by treatment with 1 μg/mL LPS for 6 h. β-Actin was used as the loading control. (**F**) Protein levels of iNOS, COX-2, and IL-1β in BV2 cells transfected with SDS3 or control siRNA, followed by treatment with 1 μg/mL LPS for 6 h. β-Actin was used as the loading control. (**G**) mRNA levels of SDS3, iNOS, COX-2, and IL-1β in BV2 cells transfected with SDS3 or Control siRNA, followed by treatment with 1 μg/mL LPS for 6 h. *ACTB* was used as the reference gene. The Control siRNA group that was not treated with LPS served as the control; *n* = 3. Error bars represent mean ± SEM. Data were analyzed by Student’s t-test; *p < 0.01; **p < 0.01; ***p < 0.001. (**H**) Concentrations of nitrite as a measure of nitric oxide production in the culture medium of BV2 cells transfected with SDS3 or Control siRNA, followed by treatment with 1 μg/mL LPS for 24 h, were determined using the Griess Reagent method; *n* = 3. Error bars represent mean ± SEM. Statistical analysis was performed using one-way analysis of variance followed by a Tukey’s multiple comparisons test; *p < 0.01. For protein band quantification information, please refer to Supplemental Fig. S1

To assess the relationship between microglial activation and the functionality of the SDS3 and Sin3/HDAC complex, we first analyzed the interaction between SDS3 and Sin3A/HDAC1 in BV2 cells via immunoprecipitation and Western blotting. We observed the protein bands of Sin3A and HDAC1 after pulldown with anti-SDS3, indicating an interaction between SDS3 and Sin3A/HDAC1 in BV2 cells (Fig. 1A).

LPS can induce downstream signaling and gene expression by binding Toll-like receptor 4 (TLR4) on the surface of microglia and is widely used in the study of molecular mechanisms underlying microglial activation and neuroinflammation. We therefore explored changes in the expression of SDS3, Sin3A, and HDAC1 in microglia after treatment with LPS.

BV2 cells were treated with 1 μg/mL LPS for different lengths of time, and changes in SDS3, Sin3A, and HDAC1 expression were examined by Western blotting and qPCR. As incubation times increased, the expression of SDS3 was significantly decreased, whereas the protein levels of HDAC1 and Sin3A did not change significantly (Fig. 1B and Supplemental Fig. S1A). This result was further validated in primary mouse microglia, where a significant downregulation in SDS3 expression was observed as LPS incubation time increased, and expression levels of Sin3A and HDAC1 did not exhibit significant changes (Supplemental Fig. S1B). To determine if LPS concentration impacts the expression of SDS3, Sin3A, and HDAC1, BV2 cells were treated with different concentrations of LPS for 6 h. SDS3 exhibited a clear decrease in expression after stimulation with 1 μg/mL LPS, whereas the expression of HDAC1 and Sin3A did not change (Fig. 1C and Supplemental Fig. S1C). Analysis of *SDS3* mRNA expression revealed similar results (Fig. 1D). Inhibition of the proteasome by treatment with MG132 followed by LPS treatment showed that inhibition of proteasome function did not affect LPS-induced downregulation of SDS3 expression (Fig. 1E), suggesting that decreased SDS3 expression is independent of proteasome-mediated protein degradation. Together, these data indicate that LPS affects SDS3 expression in BV2 cells by downregulating the expression of *SDS3* mRNA in both a time- and concentration-dependent manner, while the protein levels of Sin3A and HDAC1 remain unaffected.

### SDS3 regulates the expression of LPS-induced inflammatory factors in microglia

LPS treatment can induce the expression of different inflammatory factors in microglia, including iNOS, cyclooxygenase-2 (COX-2), and IL-1β [31]. Our experimental findings indicate that after treatment with 1 μg/mL LPS, primary mouse microglia exhibited a significant upregulation of these inflammation-related factors (Supplemental Fig. S1B). To investigate the role of SDS3 in regulating the expression of these inflammatory factors, *SDS3* was targeted using siRNA, and BV2 cells were stimulated using LPS. Following *SDS3* knockdown, the expression of iNOS, COX-2, and IL-1β was increased at both the mRNA and protein levels (Fig. 1F and G and Supplemental Fig. S1D). Accordingly, nitric oxide release, as indicated by nitrite concentration in the culture medium [32], was significantly higher in *SDS3*-knockdown cells (BV2^SDS3 − KD^) than in control cells (BV2^ctrl^) after LPS treatment (Fig. 1H).

### Analysis of SDS3 binding sites on the genome

**Fig. 2:**
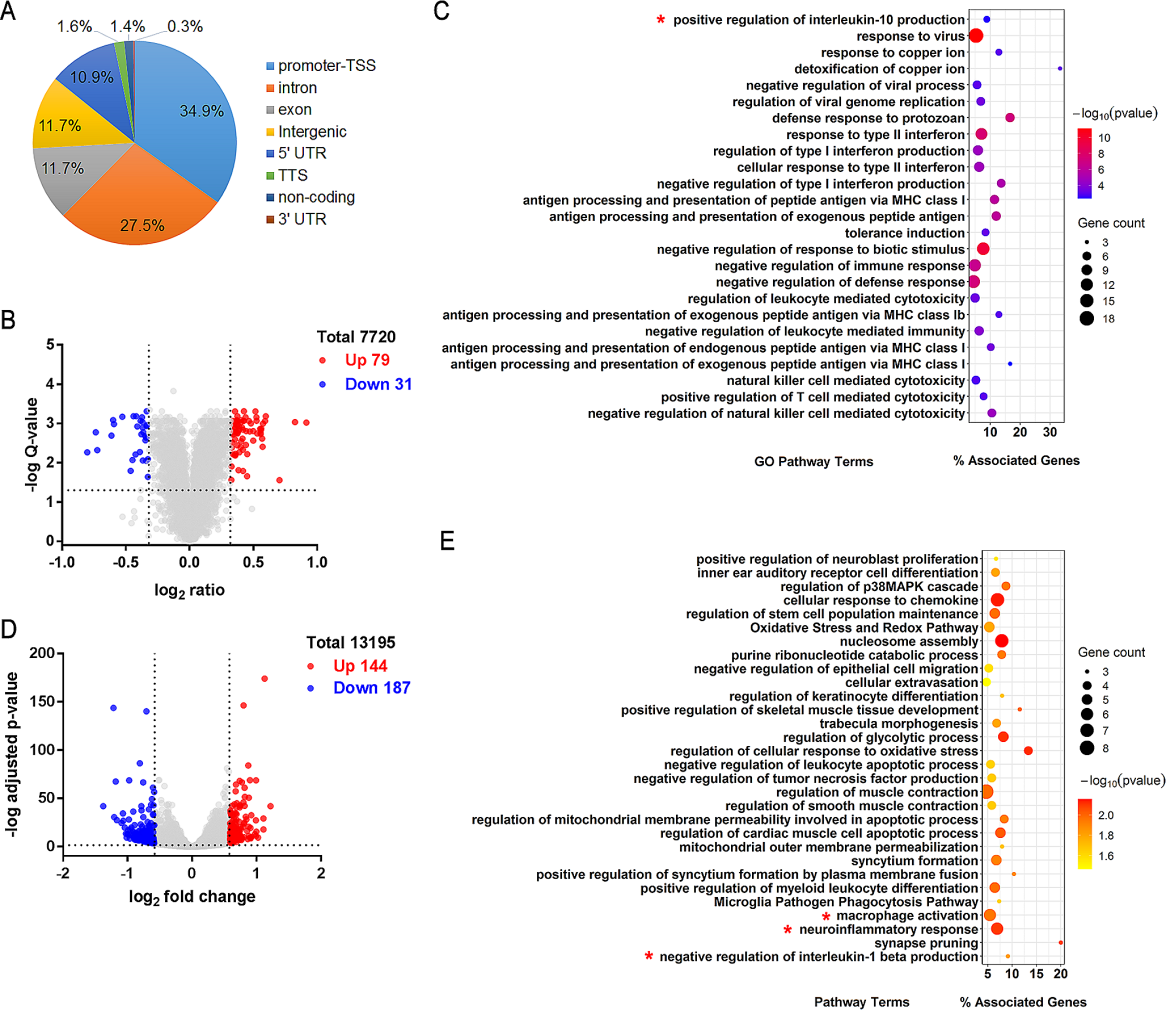
Bioinformatic enrichment analyses of differentially expressed proteins and genes in *SDS3*-knockdown BV2 cells. (**A**) Distribution of SDS3 binding sites in the genome across different gene functional elements. Chromatin immunoprecipitation with sequencing (ChIP-seq) analysis was performed to detect SDS3 binding sites in the BV2 cell genome. The ChIPseek platform was used to annotate the gene functional elements associated with these regions and calculate the percentage of each category. (**B**) Scatter plot of differentially expressed proteins in *SDS3*-knockdown cells. For proteins with ≥ 2 unique peptides, differentially expressed proteins were selected based on a *Q* value of < 0.05 and a fold change of ≥ 1.25 or ≤ 0.8. Red dots represent upregulated proteins, blue dots represent downregulated proteins, and gray dots represent unchanged proteins. (**C**) Gene Ontology (GO) enrichment analysis of differentially expressed proteins in *SDS3*-knockdown cells. Enriched bioprocess terms (*p* < 0.05) were obtained from the GO database, and the term *p* value was corrected with the Benjamini–Hochberg post hoc test. (**D**) Scatter plot of differentially expressed genes in *SDS3*-knockdown cells. Differentially expressed genes were selected based on an adjusted *p* value of < 0.05 and a fold change of ≥ 1.5 or ≤ 0.667. Red dots represent upregulated genes, blue dots represent downregulated genes, and gray dots represent unchanged genes. (**E**) Enrichment analysis of differentially expressed genes in *SDS3*-knockdown cells. Enriched terms (*p* < 0.05) were obtained from the GO Bioprocess and WikiPathways databases. The term *p* value was corrected with a Benjamini–Hochberg multiple testing correction. Terms related to inflammation-related pathways are highlighted with an asterisk (*)

To further investigate the molecular mechanisms by which SDS3 regulates inflammatory pathways in microglia, ChIP-seq was performed to analyze SDS3 binding sites across the genome. We generated a dataset of SDS3 binding sites (represented by 13,042 peaks), and the distribution of these peaks in relation to transcription start sites (TSSs) was analyzed. SDS3 binding sites were enriched near TSSs, with more than 80% of peaks located within 1 kb of a TSS (Supplemental Fig. S2A). After functional annotation of peaks using ChIPseek [33], the distribution of peaks across different gene functional elements was examined. Most peaks (34.9%) were located in the promoter and TSS regions, indicating the potential regulatory role of SDS3 (Fig. 2A). To identify SDS3 gene targets, a subset of peaks located within 1 kb upstream and 0.5 kb downstream of TSSs was selected, and the protein-coding genes associated with these peaks were identified as SDS3 targets. In total, 8,758 peaks corresponding to 7,624 genes met this criterion (Supplemental Table S3).

### SDS3 is involved in regulating inflammation-related pathways in microglia

Combined proteomic and transcriptomic analyses can be used to explore the functional mechanisms of different genes and reveal specific biological processes. To gain a more comprehensive understanding of the function of SDS3 in microglia, a quantitative proteomic analysis was performed using TMT labeling to identify protein changes in BV2 cells transfected with SDS3 or Control siRNA. Additionally, RNA-seq was used to analyze changes in transcription between BV2^SDS3 − KD^ and BV2^ctrl^ cells.

A total of 8,029 proteins were identified through mass spectrometry-based analysis, and 7,720 of these proteins had at least two unique peptides (Supplemental Fig. S2B and Table S4). Proteins that had at least two unique peptides, a *Q* value of < 0.05, and a fold change ratio of ≥ 1.25 or ≤ 0.8 were defined as differentially expressed (siSDS3_DEP). As shown in Fig. 2B, there were 110 proteins that showed significant changes in expression between BV2^SDS3 − KD^ and BV2^ctrl^ cells, with 79 proteins upregulated and 31 proteins downregulated in expression (Fig. 2B and Supplemental Table S5). GO enrichment analysis was performed on the differentially expressed proteins. Among the significantly enriched GO terms related to inflammation was “positive regulation of interleukin-10 production” (Fig. 2C).

Changes in RNA expression were assessed by RNA-seq, and 23,997 genes were identified. After multiple testing correction, 13,195 genes remained for further analysis (Supplemental Fig. S2C and Table S6), and genes with an adjusted *p* value of < 0.05 and a fold change of ≥ 1.5 or ≤ 0.667 were defined as differentially expressed (siSDS3_DEG). In total, 331 genes were significantly changed in expression after *SDS3* knockdown, with 144 upregulated and 187 downregulated (Fig. 2D, Supplemental Table S7). Subsequent GO enrichment analysis showed significantly enriched terms related to inflammation, including “neuroinflammatory response,” “macrophage activation,” and “negative regulation of interleukin-1 beta production” (Fig. 2E).

Our proteomic and transcriptomic results were not completely consistent, suggesting that post-transcriptional modifications may occur. However, both sets of results indicate the involvement of SDS3 in regulating inflammation-related pathways in microglia.

### Identification of downstream genes and regulatory pathways regulated by SDS3 in microglia

**Fig. 3:**
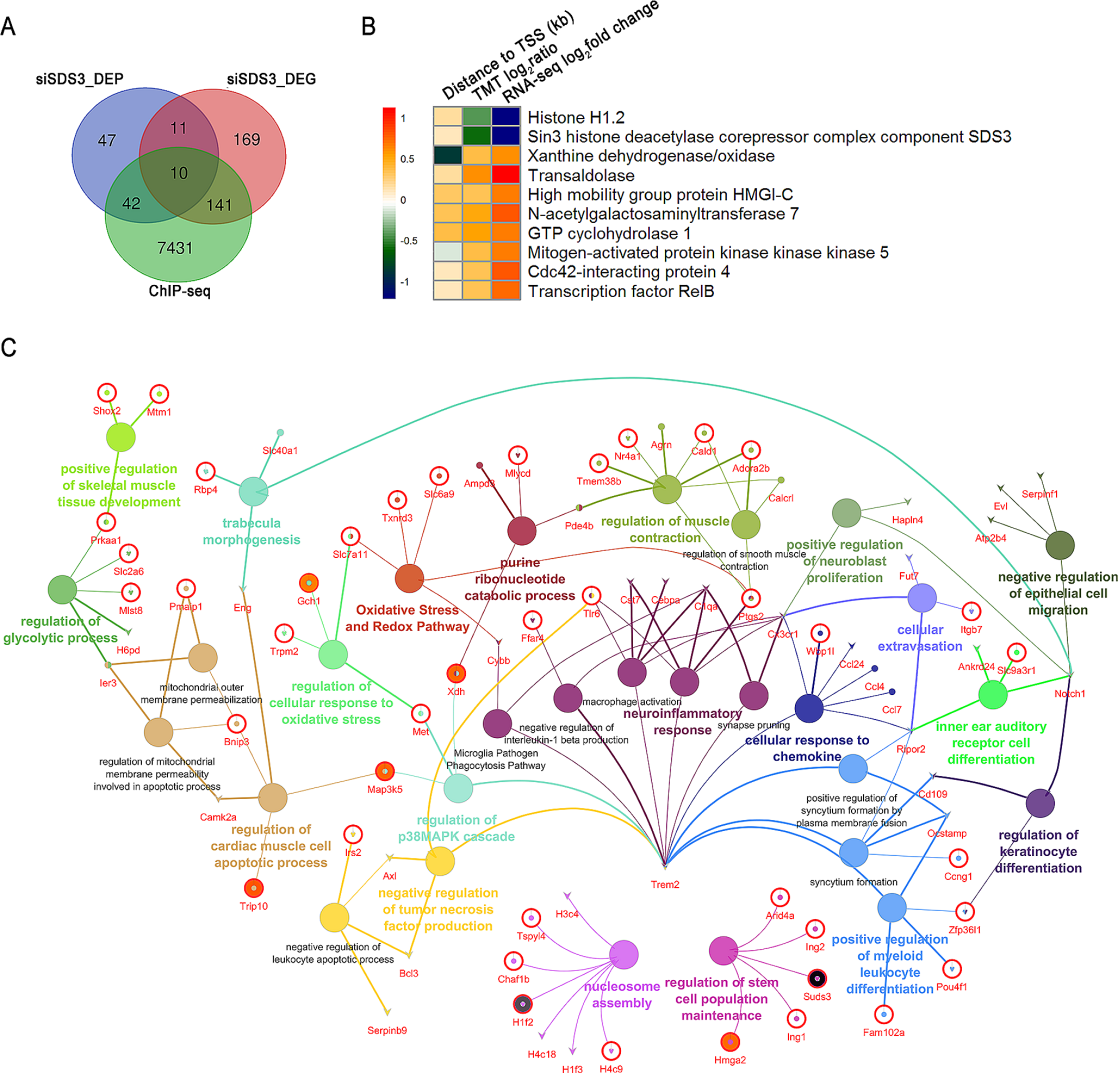
Analysis of downstream pathways regulated by SDS3. (**A**) Venn plot illustrating the overlap of genes identified by chromatin immunoprecipitation (ChIP) with sequencing, differentially expressed genes from RNA-sequencing analysis (siSDS3_DEG), and differentially expressed proteins from proteomic analysis (siSDS3_DEP). (**B**) Heat map presenting the relative ratios of the 10 overlapped genes (in terms of protein names) from RNA-sequencing and proteomic data, along with their distances to the transcription start sites (TSSs). (**C**) Network displaying the enriched bioprocess and pathway terms (*p* < 0.05) from siSDS3_DEG. The outer circle, highlighted in red, represents genes identified as SDS3 ChIP targets. The color surrounding each gene represents the ratio obtained from the proteomic data, with red indicating upregulation and blue indicating downregulation

To identify genes regulated by SDS3 in microglia, we compared SDS3 target genes identified by ChIP-seq and differentially expressed genes (siSDS3_DEG) and proteins (siSDS3_DEP) following *SDS3* knockdown. In total, 10 genes, including SDS3, were differentially expressed in both the transcriptomic and proteomic analyses (Fig. 3A) and were identified as potential SDS3 targets (Table 1; Fig. 3B). 95 genes overlapped between the upregulated genes in mRNA expression and the SDS3 target genes, suggesting that SDS3 may inhibit the expression of these 95 genes at the transcriptional level and defining them as downstream genes regulated by SDS3 (Supplemental Table S8). Additionally, 56 genes were found to be downregulated in the transcriptomic analysis and overlapped with the SDS3 target genes, indicating that SDS3 may have a transcriptional activation role for these genes. Furthermore, 180 genes showed differential expression after *SDS3* knockdown but were not identified as SDS3 target genes, suggesting that their expression may be indirectly influenced by SDS3 (Supplemental Fig. S3).

**Table 1:** Ten overlapping genes identified from RNA-seq, proteomic, and ChIP-seq data

Gene Name	Ensembl Gene	Accession	Description	TMT log2_ratio	RNA-seq log2_fold change	Distance to TSS (kb)	Annotation
Galnt7	ENSMUSG00000031608	Q80VA0	N-acetylgalactosaminyltransferase 7	0.52	0.80	0.349	intron (NR_040698, intron 1 of 3)
Hist1h1c	ENSMUSG00000036181	P15864	Histone H1.2	-0.42	-1.22	0.170	exon (NM_015786, exon 1 of 1)
Suds3	ENSMUSG00000066900	Q8BR65	Sin3 histone deacetylase corepressor complex component SDS3	-0.59	-1.18	0.105	5’ UTR (NM_178622, exon 1 of 12)
Map3k5	ENSMUSG00000071369	O35099	Mitogen-activated protein kinase kinase kinase 5	0.36	0.69	-0.116	promoter-TSS (NM_008580)
Xdh	ENSMUSG00000024066	Q00519	Xanthine dehydrogenase/oxidase	0.36	0.62	-0.871	promoter-TSS (NM_011723)
Gch1	ENSMUSG00000037580	Q05915	GTP cyclohydrolase 1	0.53	0.68	0.364	exon (NM_008102, exon 1 of 6)
Taldo1	ENSMUSG00000025503	Q93092	Transaldolase	0.60	1.13	0.163	intron (NM_011528, intron 1 of 7)
Hmga2	ENSMUSG00000056758	P52927	High mobility group protein HMGI-C	0.35	0.68	0.320	5’ UTR (NM_010441, exon 1 of 5)
Trip10	ENSMUSG00000019487	Q8CJ53	Cdc42-interacting protein 4	0.34	0.82	0.114	exon (NM_134125, exon 1 of 14)
Relb	ENSMUSG00000002983	Q04863	Transcription factor RelB	0.36	0.72	0.101	5’ UTR (NM_009046, exon 1 of 11)

ChIP-seq identified SDS3 target genes, and siSDS3_DEP were labeled on the GO enrichment analysis results of siSDS3_DEG (Fig. 3C). Genes that were increased in expression that were related to neuroinflammation, including *Ptgs2* (also known as *COX-2*, prostaglandin G/H synthase 2) and *Tlr6* (Toll-like receptor 6), as well as the downregulated gene *Ffar4* (free fatty acid receptor 4) are all SDS3 target genes. Among them, *COX-2* and *Tlr6* were transcriptionally inhibited by SDS3. *IL-1β* and *iNOS* were not identified as SDS3 target genes, suggesting that their regulation by SDS3 may be indirect. Our proteomic results showed that the change in COX-2 protein expression due to *SDS3* knockout (COX-2 ratio of 1.2) did not reach statistical significance. Similarly, in untreated BV2 cells, due to the low basal expression level of COX-2, no significant changes in COX-2 expression were observed between BV2^ctrl^ and BV2^SDS3 − KD^ cells (Fig. 1F and G). However, after LPS induction, COX-2 expression changed significantly, suggesting that the expression of COX-2 is regulated not only by SDS3 through negative regulation but also by other regulatory mechanisms.

Further analysis of the data in Fig. 3C revealed that SDS3 downstream genes, including *Map3k5*, *Met*, and *Xdh*, are involved in the regulation of the p38 MAPK signaling cascade. The corresponding protein levels of mitogen-activated protein kinase kinase kinase 5 (MAP3K5) and xanthine dehydrogenase/oxidase (XDH) were also significantly upregulated (Table 1; Fig. 3B). The p38 MAPK signaling pathway can be activated by various stimuli, both inside and outside the cell, and it is a key signaling pathway involved in the regulation of inflammatory responses [34, 35]. XDH plays a critical role in purine degradation and can influence cell activity by regulating the production of reactive oxygen species [36]. MAP3K5, also known as apoptosis signal-regulating kinase 1 (ASK1), is one of the upstream kinases of p38 MAPK. ASK1 can be activated by a range of intracellular and extracellular stimuli and, through signal transduction, activates the downstream p38 MAPK signaling pathway. SDS3 may regulate the expression of genes related to the p38 MAPK signaling pathway and thereby modulate inflammatory responses.

### SDS3 regulates the activation of p38 MAPK

Activation of p38 MAPK can be assessed by the phosphorylation of Thr180 and Tyr182 (referred to as p-p38). In BV2 cells, *SDS3* knockdown was performed, and changes in p-p38 before and after LPS treatment were examined. In the absence of LPS treatment, *SDS3* knockdown led to an upregulation in p-p38 compared with that observed in the control group (Control siRNA). After LPS treatment, there was a significant increase in p-p38 in microglia, and *SDS3* knockdown further enhanced this process, resulting in the highest level of p-p38. This suggests that *SDS3* knockdown promotes activation of the p38 MAPK pathway in microglia.

**Fig. 4:**
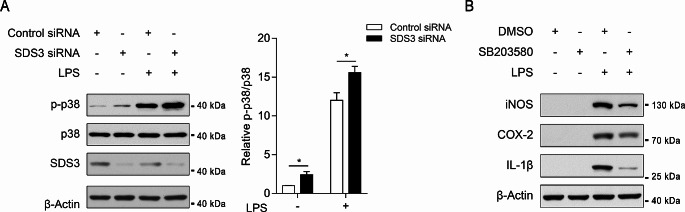
SDS3 knockdown enhances lipopolysaccharide (LPS)-induced p38 MAPK activation. (**A**) BV2 cells transfected with SDS3 or Control siRNA were treated with 1 μg/mL LPS for 1 h to assess p38 phosphorylation. The Control siRNA group served as the control group; *n* = 3. Error bars represent mean ± SEM. Data were analyzed by Student’s t-test; *p < 0.05. (**B**) BV2 cells were treated with 20 μM SB203580 (p38 MAPK inhibitor) or DMSO for 1 h, followed by treatment with 1 μg/mL LPS for 6 h, and protein levels of iNOS, COX-2, and IL-1β were measured. β-Actin was used as the internal reference protein. For protein band quantification information, refer to Supplemental Fig. S4A

SB203580 is an inhibitor of p38 MAPK kinase activity that negatively regulates the p38 MAPK signaling pathway and its downstream effects. We treated BV2 cells with SB203580 or DMSO followed by LPS and assessed the expression of inflammatory factors by Western blotting. Inhibiting p38 MAPK activity resulted in decreased expression of LPS-induced inflammatory factors (Fig. 4B and Supplemental Fig. S4A, quantitative bar graph), indicating that the p38 MAPK signaling pathway can regulate the LPS-induced inflammatory response in microglia.

### SDS3 regulates the expression of ASK1

**Fig. 5:**
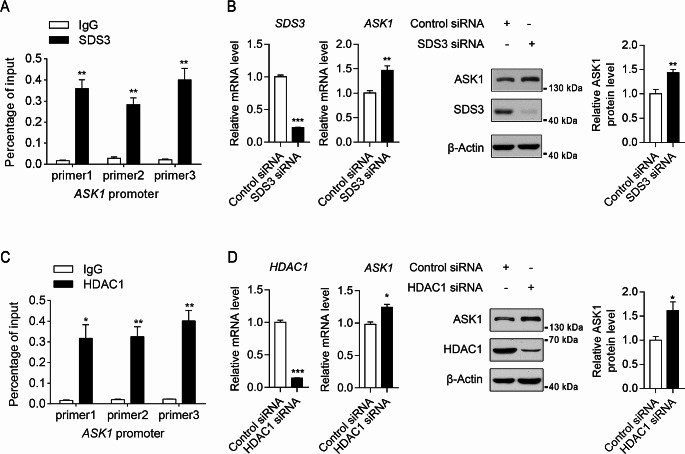
Regulation of ASK1 expression by SDS3 and HDAC1. (**A**) Binding of SDS3 to the promoter region of *ASK1*. ChIP experiments were performed using anti-SDS3 or negative-control IgG on chromatin fractions from formaldehyde cross-linked BV2 cells. Quantitative PCR (qPCR) analysis was conducted to assess the binding of SDS3 to the promoter region of *ASK1*. Results are presented as a percentage of input. IgG was used as the control group; *n* = 3. Error bars represent mean ± SEM. Data were analyzed by Student’s t-test; **p < 0.01. (**B**) Upregulation of ASK1 expression after *SDS3* knockdown. BV2 cells were transfected with SDS3 or Control siRNA, and changes in SDS3 and ASK1 mRNA and protein expression were measured. β-Actin was used as the internal reference. The Control siRNA group served as the control group; *n* = 3. Error bars represent mean ± SEM. Data were analyzed by Student’s t-test; *p < 0.05; **p < 0.01; ***p < 0.001. (**C**) Binding of HDAC1 to the *ASK1* promoter. ChIP experiments were performed using anti-HDAC1 or negative-control IgG on chromatin fractions from formaldehyde cross-linked BV2 cells. qPCR analysis was conducted to assess the binding of HDAC1 to the *ASK1* promoter region. Results are presented as a percentage of input. IgG was used as the control group; *n* = 3. Error bars represent mean ± SEM. Data were analyzed by Student’s t-test; *p < 0.05; **p < 0.01. (D) Upregulation of ASK1 expression after HDAC1 knockdown. BV2 cells were transfected with HDAC1 or Control siRNA, and changes in HDAC1 and ASK1 mRNA and protein expression were measured. β-Actin was used as the internal reference. The Control siRNA group served as the control group; *n* = 3. Error bars represent mean ± SEM. Data were analyzed by Student’s t-test; *p < 0.05; **p < 0.01; ***p < 0.001

To validate the transcriptional regulation of ASK1 by SDS3, three primer pairs were designed in the *ASK1* promoter approximately 0, 500, and 800 bp upstream of the first exon. ChIP experiments were then conducted in BV2 cells using anti-SDS3 or IgG control antibody to enrich DNA fragments bound by SDS3 and assess the DNA content of the *ASK1* promoter by qPCR, thereby determining the ability of SDS3 to bind the *ASK1* promoter. Pulldown with anti-SDS3 demonstrated enrichment of the *ASK1* promoter region compared with pulldown with IgG control (Fig. 5A), indicating that SDS3 can bind to the *ASK1* promoter. *SDS3* knockdown was then performed in BV2 cells, and the expression of ASK1 was examined. Consistent with the proteomic and RNA-seq results, *SDS3* knockdown resulted in a significant upregulation in ASK1 expression (Fig. 5B), indicating that *SDS3* knockdown can enhance the expression of ASK1 in microglia, thus confirming *ASK1* as a downstream gene that is negatively regulated by SDS3.

In addition, SDS3 forms a complex with HDAC1 in microglia (Fig. 1A), and ChIP-qPCR experiments showed that the *ASK1* promoter region is enriched following pulldown with anti-HDAC1 (Fig. 5C). Similarly, knockdown of *HDAC1* in BV2 cells also increased ASK1 expression (Fig. 5D). These results suggest that HDAC1 also binds to the *ASK1* promoter region, indicating that SDS3 may inhibit the expression of ASK1 by forming the Sin3/HDAC transcriptional co-repressor complex.

### ASK1 regulates microglial inflammatory processes through the p38 MAPK signaling pathway

**Fig. 6:**
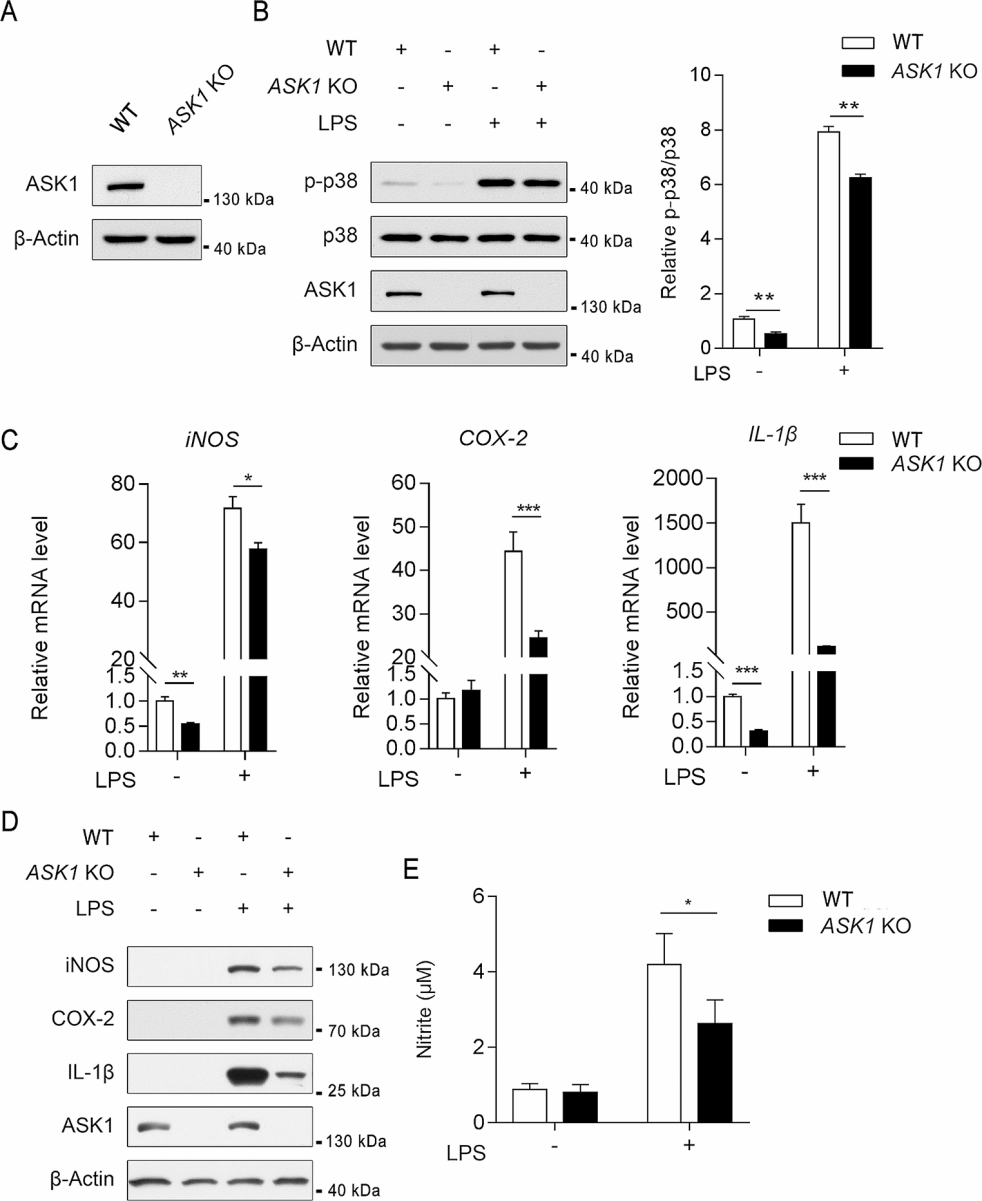
ASK1 knockout inhibits LPS-induced p38 MAPK activation and downregulates the expression of LPS-induced inflammatory factors. (**A**) Protein expression levels of ASK1 in *ASK1*-knockout monoclonal cells (*ASK1*-KO) and control cells (WT). (**B**) Inhibition of LPS-induced p38 MAPK activation by *ASK1* knockout. *ASK1*-KO and WT BV2 cells were treated with 1 μg/mL LPS for 1 h, and p38 phosphorylation was detected. Statistical analysis was performed on the relative levels of p38 phosphorylation, with the WT group (untreated with LPS) as the reference group; *n* = 3. Error bars represent mean ± SEM. Data were analyzed by Student’s t-test; **p < 0.01. (**C**) Downregulation of inflammatory factor gene expression after *ASK1* knockout. *ASK1*-KO and WT BV2 cells were treated with 1 μg/mL LPS for 6 h, and the mRNA levels of iNOS, COX-2, and IL-1β were measured. *ACTB* was used as the reference gene, with the untreated WT BV2 as the reference group; *n* = 3. Error bars represent mean ± SEM. Data were analyzed by Student’s t-test; *p < 0.05; ***p < 0.001. (**D**) Downregulation of inflammatory factor protein expression by *ASK1* knockout. *ASK1*-KO and WT BV2 cells were treated with 1 μg/mL LPS for 6 h, and the protein levels of iNOS, COX-2, and IL-1β were measured. Protein band quantification information can be found in Supplemental Fig. S4B. (**E**) Reduction of nitric oxide generation by *ASK1* knockout. *ASK1*-KO and WT BV2 cells were treated with 1 μg/mL LPS for 24 h, and the concentration of nitrite in the culture medium was measured using the Griess Reagent method to assess nitric oxide production; *n* = 3. Error bars represent mean ± SEM. Data were analyzed by one-way analysis of variance and a Tukey’s multiple comparisons post hoc test; *p < 0.05

ASK1, as an upstream kinase of the p38 MAPK, plays a crucial role in regulating the inflammatory response in microglia. To investigate the role of ASK1 in inflammation, *ASK1* was knocked out using CRISPR-Cas9 technology in BV2 cells (BV2^ASK1 − KO^; Fig. 6A). Compared with normal cells (BV2^WT^), p-p38 was significantly decreased in BV2^ASK1 − KO^ cells (Fig. 6B). After LPS stimulation, p-p38 levels were significantly increased in both cell groups; however, p-p38 was lower in BV2^ASK1 − KO^ cells than in BV2^WT^ cells (Fig. 6B). Following LPS stimulation, the expression of iNOS, COX-2, and IL-1β was significantly upregulated in both BV2^WT^ and BV2^ASK1 − KO^ cells compared with untreated cells; however, expression in BV2^ASK1 − KO^ cells was lower than in BV2^WT^ cells (Fig. 6C and D and Supplemental Fig. S4B, quantitative bar graph). Accordingly, BV2^WT^ and BV2^ASK1 − KO^ cells exhibited an increase in nitric oxide production after LPS treatment; however, nitric oxide production in BV2^ASK1 − KO^ cells was lower than in BV2^WT^ cells (Fig. 6E). Together, these results suggest that knocking out *ASK1* can partially inhibit activation of p38 MAPK induced by LPS, thereby reducing the expression of the inflammatory factors iNOS, COX2, and IL-1β and inhibiting nitric oxide production in microglia.

### *ASK1* knockout affects the regulation of SDS3 and the inflammatory response

**Fig. 7:**
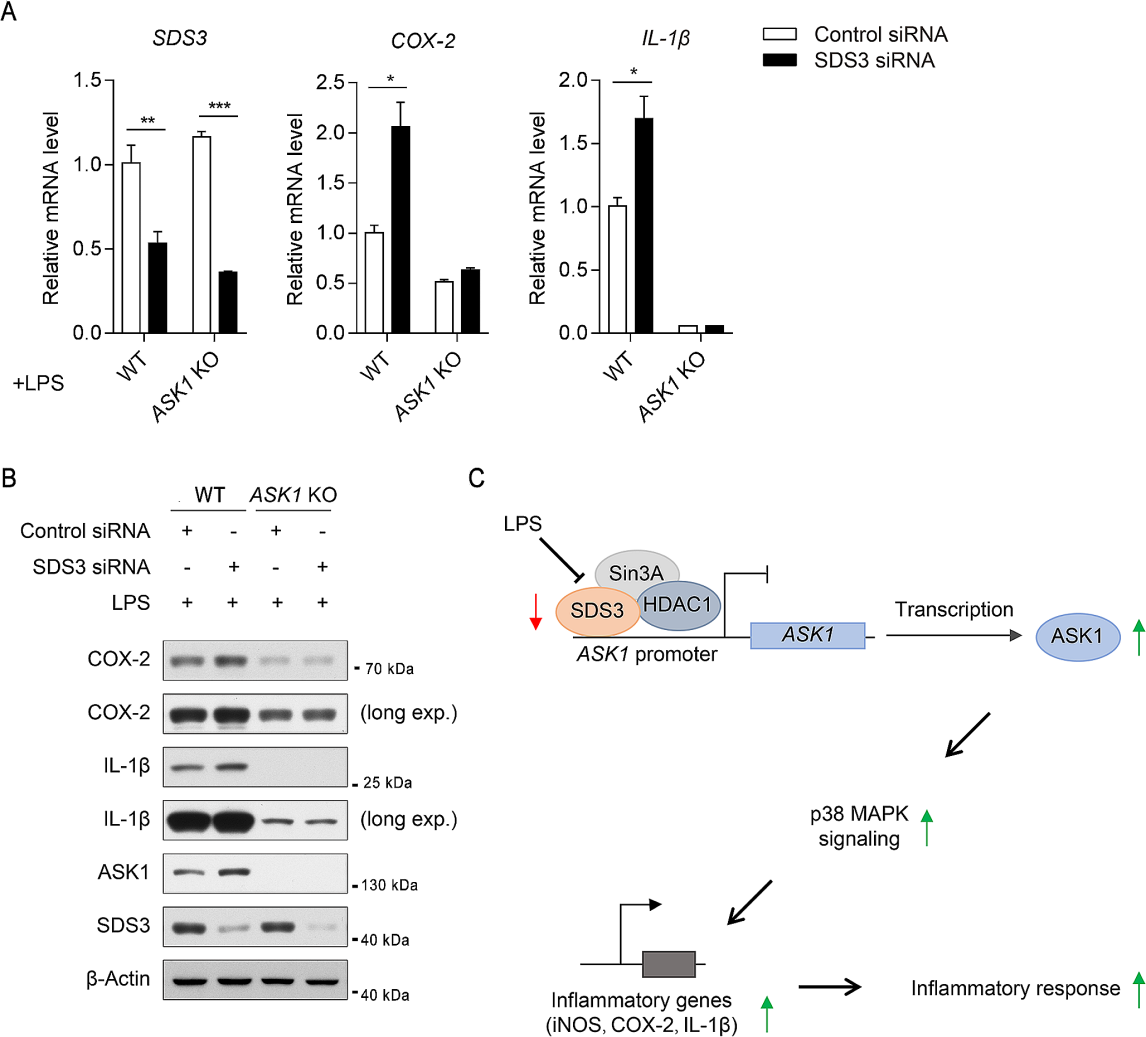
Analysis of SDS3 regulation of the inflammatory response after *ASK1* knockout. (**A**) In *ASK1*-KO cells, SDS3 no longer regulates the transcription of inflammatory factors. *ASK1* KO and WT BV2 cells were transfected with SDS3 or Control siRNA, followed by treatment with 1 μg/mL LPS for 6 h. The mRNA expression levels of SDS3, COX-2, and IL-1β were measured, and *ACTB* was used as the internal reference gene. WT BV2 cells transfected with Control siRNA represents the control group; *n* = 3. Error bars represent mean ± SEM. Data were analyzed by Student’s t-test; *p < 0.05; **p < 0.01; ***p < 0.001. (**B**) After knocking out *ASK1*, SDS3 no longer regulates the protein expression of inflammatory factors. *ASK1*-KO and WT BV2 cells were transfected with SDS3 or Control siRNA, followed by treatment with 1 μg/mL LPS for 6 h. The protein levels of SDS3, COX-2, and IL-1β were measured. “Long exp” indicates a long exposure of the same band. Protein band quantification information can be found in Supplemental Fig. S4C. (**C**) Schematic of the molecular mechanism of SDS3 regulation in the microglial inflammatory response

LPS stimulation downregulates SDS3 expression (Fig. 1), and knockdown of *SDS3* can upregulate the expression of LPS-induced inflammatory factors (Fig. 2). To investigate whether *ASK1* knockout affects the regulation of SDS3 and the expression of inflammatory factors, BV2^ASK1 − KO^ and BV2^WT^ cells were transfected with SDS3 siRNA (SDS3-KD) or Control siRNA (ctrl). After 6 h of LPS treatment, compared with the control group (BV2^WT + ctrl^), the expression of SDS3 was significantly downregulated in BV2^WT + SDS3−KD^ cells, indicating successful knockdown of SDS3 expression (Fig. 7A and B). Moreover, the expression of COX-2 and IL-1β was significantly upregulated in BV2^WT + SDS3−KD^ cells compared to in BV2^WT + ctrl^ cells, indicating that knocking down *SDS3* significantly increases the expression of COX-2 and IL-1β in BV2^WT + SDS3−KD^ cells (Fig. 7A and B). In BV2^ASK1 − KO^ cells, after LPS treatment, SDS3 expression was similarly decreased in BV2^ASK1 − KO + SDS3−KD^ and BV2^ASK1 − KO + ctrl^ cells. However, because of a lack of ASK1 in these cells, there were no significant changes in the expression of COX-2 and IL-1β between BV2^ASK1 − KO + SDS3−KD^ and BV2^ASK1 − KO + ctrl^ cells even after LPS stimulation (Fig. 7A and B and Supplemental Fig. S4C, quantitative bar graph). These results indicate that knocking out *ASK1* affects the regulation of SDS3 on the expression of inflammatory mediators. Additionally, the regulation of microglial inflammation by SDS3, to some extent, depends on its inhibition of the downstream gene *ASK1*.

## Discussion

LPS is a major component of the outer membrane of Gram-negative bacteria and is one of the most extensively studied ligands for TLR4, a typical pattern recognition receptor involved in initiating infectious and noninfectious inflammatory responses [37, 38]. TLR4 is expressed in neurons and glia in the central nervous system, with the highest expression levels observed in microglia [16, 39]. LPS can activate microglia, leading to the production of proinflammatory cytokines and inflammatory mediators, such as nitric oxide, which can cause neuronal dysfunction and death [40, 41]. Therefore, LPS is widely used in molecular studies investigating microglial activation and neuroinflammation related to neurodegeneration [31, 42, 43].

In this study, we validated that SDS3 interacts with Sin3A and HDAC1 proteins in microglia (Fig. 1A) and found that the expression of SDS3 was decreased after LPS stimulation, and this decrease was not the result of proteasomal degradation (Fig. 1B–E). Other components of the Sin3/HDAC complex did change in expression. Contradictory data exist regarding the role of HDAC1/2 in inflammation [44–47]. This may be due to the lack of specificity of the HDAC inhibitors used, which cannot precisely regulate the activity of HDAC1/2. Alternatively, HDAC1/2 can form various complexes and exert complex regulatory functions. Similarly, Sin3A, as a scaffold protein of the complex, participates in the formation of the Sin3/HDAC complex and also interacts with various components of histone modification complexes and transcription factors, exhibiting complex gene regulatory functions [48]. As SDS3 is a regulatory subunit of the Sin3/HDAC complex, it can mediate the simultaneous deacetylation of distantly located nucleosomes by forming dimers and increase the chromatin anchoring ability of the complex by binding to DNA, which is necessary for the integrity and catalytic activity of the Sin3/HDAC complex [26]. SDS3 may act as a “switch” molecule, mediating flexible regulation of the recruitment sites and catalytic activity of the complex. Previous reports have shown that the expression of regulatory subunits can modulate the function of histone modification complexes [49]. Compared with Sin3A and HDAC1, SDS3 has a simpler and more specific molecular interaction in the complex. Therefore, interfering with SDS3 to explore the function of the Sin3/HDAC complex in the inflammatory response is more specific and straightforward.

In macrophages, the Sin3A/HDAC complex can inhibit the expression of proinflammatory genes by binding to their promoter regions, thus suppressing the inflammatory response [23, 24]. Here, we demonstrated that knocking down *SDS3* in BV2 cells can promote the expression of LPS-induced inflammatory factors (iNOS, COX-2, and IL-1β) and the release of nitric oxide (Fig. 1F-H). By integrating transcriptomic and proteomic data and conducting bioinformatic analyses, we explored changes in mRNA and protein profiles in *SDS3*-knockdown microglia. Based on a clustering analysis of differentially expressed genes and proteins, we hypothesized that SDS3 is involved in regulating biological processes related to microglial inflammation (Fig. 2C and E), and our results support this hypothesis. ChIP-seq analysis combined with transcriptomics identified genes potentially regulated by SDS3 (Supplemental Table S8). Analysis of the biological pathways in which these downstream genes are involved revealed the participation of SDS3-regulated genes in the p38 MAPK signaling pathway (Fig. 3C). Subsequent experiments in BV2 cells showed that knocking down *SDS3* can upregulate p-p38, suggesting activation of the p38 MAPK signaling pathway. LPS stimulation increased p-p38 in BV2 cells, and knocking down *SDS3* further enhanced this phosphorylation (Fig. 4A), confirming the negative regulation of SDS3 on the p38 MAPK signaling pathway. These findings indicate that SDS3 plays an important role in regulating the inflammatory response through modulation of the p38 MAPK signaling pathway.

MAPKs are a group of serine and threonine kinases that can translate extracellular stimuli into a series of intracellular responses. Activated p38 MAPK can phosphorylate a range of nuclear and cytoplasmic proteins primarily involved in gene transcription regulation [50, 51]. The p38 MAPK signaling pathway is rapidly activated in response to various stress conditions, such as oxidative stress, osmotic stress, endoplasmic reticulum stress, and inflammatory stimuli, and it regulates cellular processes to adapt to these stimuli [52]. Inhibiting p38 activity reduces the production of IL-8 under osmotic stress [53] and IL-6 under TNFα stimulation [54]. In addition, inhibiting p38 activity hinders the expression of COX-2 in macrophages stimulated by LPS [55], and in p38-deficient macrophages, the production of TNFα, IL-12, and IL-18 induced by LPS is reduced [56], indicating that inhibiting p38 MAPK activity can decrease the production of inflammatory factors. In microglia, LPS can activate the p38 MAPK signaling pathway and promote the production of TNFα, IL-1β, and nitric oxide [57, 58]. Additionally, the Aβ25–35 fragment activates the p38 MAPK signaling pathway and induces the generation of IL-1β and nitric oxide in microglia, whereas fibrillar Aβ1–42 induces the production of TNFα and IL-1β through the p38 MAPK pathway [59]. Following ischemic brain injury, disruption of the blood–brain barrier and neuronal cell death lead to the exposure to substances in blood vessels and within brain cells, which can activate microglia and trigger p38 MAPK pathway activation, leading to the production of inflammatory factors [60–62]. Therefore, under pathological conditions, such as ND and brain injury, activation of the p38 MAPK signaling pathway in microglia promotes neuroinflammation and exacerbates disease progression. Our experiments also confirmed that inhibiting p38 MAPK activity in BV2 cells decreases the expression of LPS-induced inflammatory factors (Fig. 4B), validating the regulatory role of the p38 MAPK pathway in the inflammatory response.

Our analysis revealed that *ASK1* and *XDH*, the downstream genes of SDS3, are involved in regulating the p38 MAPK signaling pathway. We observed that the expression of ASK1 and XDH was upregulated in BV2^SDS3 − KD^ cells (Fig. 3B; Table 1). ASK1 is a MAP3K protein that directly phosphorylates MKK3/MKK6, which are specific MAP2Ks of the p38 MAPK family. MKK3/MKK6, in turn, directly phosphorylate the threonine and tyrosine residues of the Thr-Gly-Tyr motif, activating the downstream p38 signaling pathway and participating in the regulation of inflammatory responses under various stimuli [63, 64]. The kinase activity and abundance of ASK1 are regulated by multiple mechanisms, with previous studies primarily focusing on post-translational modifications and protein degradation [65–69]. Less is known about the regulatory mechanisms of ASK1 expression. Among the known proteins that regulate ASK1 expression, E2F1, KLF5, and HNF4α promote expression, whereas BRG1 inhibits it [66, 67, 70, 71]. Our ChIP-qPCR experiments demonstrated that both SDS3 and HDAC can bind to the *ASK1* promoter, and knocking down *SDS3* or *HDAC* can promote the expression of ASK1 (Fig. 5). This suggests that SDS3 inhibits the expression of ASK1 through the formation of transcriptional complexes in microglia. These findings reveal new proteins involved in the regulation of ASK1 expression.

ASK1 is involved in the activation of signaling pathways, including the NF-κB and MAPK signaling pathways, which are mediated by TLRs, and plays a role in the natural immune response, including inflammation. For example, in splenocytes and dendritic cells from *ASK1*-knockout mice stimulated with the TLR4 ligand LPS, the production of proinflammatory cytokines, such as TNFα, IL-6, and IL-1β, was reduced, and activation of the p38 MAPK pathway was inhibited [72]. Similarly, in 293T cells overexpressing the TLR2 receptor, activation of the p38 MAPK signaling pathway was observed after ligand stimulation, whereas overexpression of a dominant-negative form of ASK1 significantly inhibited activation of the p38 MAPK signaling pathway [73]. Consistently, macrophages overexpressing the dominant-negative form of ASK1 showed a significant reduction in TNFα and IL-6 production after TLR2 ligand stimulation [74, 75]. These studies suggest that ASK1 mediates inflammation through the p38 MAPK signaling pathway. Additionally, experiments in *ASK1*-knockout mice showed that ASK1 promotes inflammatory responses in diseases such as allergic asthma, rheumatoid arthritis, drug-induced liver injury, and contact hypersensitivity [76]. In microglia, ASK1 is involved in regulating cellular responses to various external stimuli. For example, microglia from *ASK1*-knockout mice showed reduced production of TNFα and iNOS after LPS stimulation [77]. In the brains of ASK1-deficient mice, decreased activation of microglia and reduced levels of TNFα, IL-6, and IL-1β were observed following brain ischemia. Similarly, knocking down *ASK1* in BV2 cells led to a decrease in proinflammatory cytokine production induced by oxygen-glucose deprivation [78]. Stimulation with high glucose induced upregulation of ASK1 expression in BV2 cells, and inhibition of ASK1 activity resulted in decreased expression of TNFα and IL-6 induced by high glucose [79]. Furthermore, ASK1 promotes cobalt protoporphyrin-induced COX-2 expression in BV2 cells [80]. These studies collectively indicate that ASK1 is involved in promoting inflammatory responses in microglia after stimulation by various factors.

Our experiments revealed that knocking out *ASK1* in BV2 cells leads to a decrease in p-p38 and inhibition of the expression of LPS-induced inflammatory factors (Fig. 6), indicating that ASK1 can mediate LPS-induced inflammatory responses through the regulation of the p38 MAPK signaling pathway, which is consistent with previous findings. In BV2 *ASK1*-knockout cells, knocking down *SDS3* expression did not affect the expression of certain LPS-induced inflammatory factors (Fig. 7), further suggesting that ASK1 is a necessary downstream protein for the inflammatory regulatory role of SDS3.

In summary, we discovered that LPS stimulation downregulates the expression of the transcriptional co-repressor SDS3 in microglia, and SDS3 regulates the activation of the p38 MAPK signaling pathway through the modulation of the upstream kinase ASK1, thereby modulating LPS-induced inflammatory responses in microglia (Fig. 7C). It is worth noting that SDS3 does not possess specific DNA-binding activity, and its recruitment to the genome depends on sequence-specific transcription factors such as FOXK1 [81]. Further studies are needed to investigate the specific transcription factors that can bind to the *ASK1* promoter region and the involvement of other coacting histone-modifying enzymes in the regulation of *ASK1* expression.

## Conclusion

Neuroinflammation is a stress response in the central nervous system that helps maintain homeostasis. Excessive and persistent neuroinflammation leads to neuron death and contributes to the pathogenesis of various NDs. Microglia play a crucial role in maintaining central nervous system homeostasis. Excessive and sustained neuroinflammation mediated by microglia leads to neuron dysfunction and death, which is closely associated with the pathogenesis of various NDs. The Sin3/HDAC complex is a classic multiprotein complex in mammals that is primarily involved in transcriptional repression through its deacetylase activity. SDS3 is a core component of the Sin3/HDAC complex and is essential for maintaining complex integrity and deacetylase activity. Through a series of experiments and ‘omics analyses, we validated that downregulation of SDS3 can promote the expression of the upstream kinase ASK1, activate the p38 MAPK signaling pathway, and subsequently enhance LPS-induced inflammatory responses in microglia. We provide important evidence of the contributions of SDS3 toward microglial inflammation, thereby extending the inhibitory role of the Sin3/HDAC complex to inflammation in microglia. Importantly, our findings highlight the crucial role of SDS3 in this regulatory function and provide new insights into the biological function of SDS3 and the regulatory mechanisms of microglial inflammation.

### Electronic supplementary material

Below is the link to the electronic supplementary material.

Supplementary Material 1

Supplementary Material 2

Supplementary Material 3

Supplementary Material 4

Supplementary Material 5

Supplementary Material 6

Supplementary Material 7

Supplementary Material 8

Supplementary Material 9

## Data Availability

No datasets were generated or analysed during the current study.

## References

[1] Wood LB, Winslow AR, Strasser SD. Systems biology of neurodegenerative diseases. Integr Biol. 2015;7:758–75. 10.1039/C5IB00031A PMC4587766 26065845

[2] Dugger BN, Dickson DW. Pathology of neurodegenerative diseases. Cold Spring Harb Perspect Biol. 2017;9:a028035. 28062563 10.1101/cshperspect.a028035 PMC5495060

[3] Ransohoff RM, Schafer D, Vincent A, Blachère NE, Bar-Or A. Neuroinflammation: ways in which the immune system affects the brain. Neurotherapeutics. 2015;12:896–909. 26306439 10.1007/s13311-015-0385-3 PMC4604183

[4] Ransohoff RM. How neuroinflammation contributes to neurodegeneration. Science. 2016;353:777–83. 27540165 10.1126/science.aag2590

[5] Lambert J-C, Heath S, Even G, Campion D, Sleegers K, Hiltunen M, et al. Genome-wide association study identifies variants at CLU and CR1 associated with Alzheimer’s disease. Nat Genet. 2009;41:1094. 19734903 10.1038/ng.439

[6] Hollingworth P, Harold D, Sims R, Gerrish A, Lambert J-C, Carrasquillo MM, et al. Common variants at ABCA7, MS4A6A/MS4A4E, EPHA1, CD33 and CD2AP are associated with Alzheimer’s disease. Nat Genet. 2011;43:429. 21460840 10.1038/ng.803 PMC3084173

[7] Naj AC, Jun G, Beecham GW, Wang L-S, Vardarajan BN, Buros J, et al. Common variants at MS4A4/MS4A6E, CD2AP, CD33 and EPHA1 are associated with late-onset Alzheimer’s disease. Nat Genet. 2011;43:436–41. 21460841 10.1038/ng.801 PMC3090745

[8] Jonsson T, Stefansson H, Steinberg S, Jonsdottir I, Jonsson PV, Snaedal J, et al. Variant of TREM2 associated with the risk of Alzheimer’s disease. N Engl J Med. 2013;368:107–16. 23150908 10.1056/NEJMoa1211103 PMC3677583

[9] Hamza TH, Zabetian CP, Tenesa A, Laederach A, Montimurro J, Yearout D, et al. Common genetic variation in the HLA region is associated with late-onset sporadic Parkinson’s disease. Nat Genet. 2010;42:781. 20711177 10.1038/ng.642 PMC2930111

[10] Kauwe JSK, Bailey MH, Ridge PG, Perry R, Wadsworth ME, Hoyt KL et al. Genome-wide association study of CSF levels of 59 alzheimer’s disease candidate proteins: significant associations with proteins involved in amyloid processing and inflammation. PLoS Genet. 2014;10. 10.1371/journal.pgen.1004758 PMC4207667 25340798

[11] Daniele SG, Béraud D, Davenport C, Cheng K, Yin H, Maguire-Zeiss KA. Activation of MyD88-dependent TLR1/2 signaling by misfolded α-synuclein, a protein linked to neurodegenerative disorders. Sci Signal. 2015;8:ra45–45. 25969543 10.1126/scisignal.2005965 PMC4601639

[12] Kim C, Ho D-H, Suk J-E, You S, Michael S, Kang J, et al. Neuron-released oligomeric α-synuclein is an endogenous agonist of TLR2 for paracrine activation of microglia. Nat Commun. 2013;4:1562. 23463005 10.1038/ncomms2534 PMC4089961

[13] El Khoury JB, Moore KJ, Means TK, Leung J, Terada K, Toft M, et al. CD36 mediates the innate host response to β-amyloid. J Exp Med. 2003;197:1657–66. 12796468 10.1084/jem.20021546 PMC2193948

[14] Stewart CR, Stuart LM, Wilkinson K, van Gils JM, Deng J, Halle A, et al. CD36 ligands promote sterile inflammation through assembly of a toll-like receptor 4 and 6 heterodimer. Nat Immunol. 2010;11:155–61. 20037584 10.1038/ni.1836 PMC2809046

[15] Ransohoff RM, Perry VH. Microglial physiology: unique stimuli, specialized responses. Annu Rev Immunol. 2009;27:119–45. 19302036 10.1146/annurev.immunol.021908.132528

[16] Labzin LI, Heneka MT, Latz E. Innate immunity and neurodegeneration. Annu Rev Med. 2018;69:437–49. 29106805 10.1146/annurev-med-050715-104343

[17] Millard CJ, Watson PJ, Fairall L, Schwabe JWR. Targeting class I histone deacetylases in a complex environment. Trends Pharmacol Sci. 2017;38:363–77. 28139258 10.1016/j.tips.2016.12.006

[18] Grandinetti KB, Jelinic P, DiMauro T, Pellegrino J, Fernandez Rodriguez R, Finnerty PM, et al. Sin3B expression is required for cellular senescence and is up-regulated upon oncogenic stress. Cancer Res. 2009;69:6430–7. 19654306 10.1158/0008-5472.CAN-09-0537 PMC2782780

[19] Dannenberg JH. mSin3A corepressor regulates diverse transcriptional networks governing normal and neoplastic growth and survival. Genes Dev. 2005;19:1581–95. 15998811 10.1101/gad.1286905 PMC1172064

[20] van Oevelen C, Wang J, Asp P, Yan Q, Kaelin WG, Kluger Y, et al. A role for mammalian Sin3 in permanent gene silencing. Mol Cell. 2008;32:359–70. 18995834 10.1016/j.molcel.2008.10.015 PMC3100182

[21] Kadosh D, Struhl K. Targeted recruitment of the Sin3-Rpd3 histone deacetylase complex generates a highly localized domain of repressed chromatin in vivo. Mol Cell Biol. 1998;18:5121–7. 9710596 10.1128/MCB.18.9.5121 PMC109097

[22] Kim M, Lu F, Zhang Y. Loss of HDAC-mediated repression and gain of NF-κB activation underlie cytokine induction in ARID1A-and PIK3CA-mutation-driven ovarian cancer. Cell Rep. 2016;17:275–88. 27681437 10.1016/j.celrep.2016.09.003 PMC7734570

[23] Hwang S-Y, Hwang J-S, Kim S-Y, Han I-O. O-GlcNAc transferase inhibits LPS-mediated expression of inducible nitric oxide synthase through an increased interaction with mSin3A in RAW264. 7 cells. Am J Physiology-Cell Physiol. 2013;305:C601–8. 10.1152/ajpcell.00042.2013 23824843

[24] John SP, Sun J, Carlson RJ, Cao B, Bradfield CJ, Song J, et al. IFIT1 exerts opposing regulatory effects on the inflammatory and interferon gene programs in LPS-activated human macrophages. Cell Rep. 2018;25:95–e1066. 30282041 10.1016/j.celrep.2018.09.002 PMC6492923

[25] Alland L, David G, Shen-Li H, Potes J, Muhle R, Lee HC, et al. Identification of mammalian Sds3 as an integral component of the Sin3/histone deacetylase corepressor complex. Mol Cell Biol. 2002;22:2743–50. 11909966 10.1128/MCB.22.8.2743-2750.2002 PMC133736

[26] Clark MD, Marcum R, Graveline R, Chan CW, Xie T, Chen Z, et al. Structural insights into the assembly of the histone deacetylase-associated Sin3L/Rpd3L corepressor complex. PNAS. 2015;112:E3669–78. 26124119 10.1073/pnas.1504021112 PMC4507224

[27] Zhang K, Dai X, Wallingford MC, Mager J. Depletion of Suds3 reveals an essential role in early lineage specification. Dev Biol. 2013;373:359–72. 23123966 10.1016/j.ydbio.2012.10.026

[28] Lai W, Li D, Wang Q, Ma Y, Tian J, Fang Q. Bacterial magnetosomes release iron ions and induce regulation of iron homeostasis in endothelial cells. Nanomaterials (Basel, Switzerland). 2022;12. 10.3390/nano12223995 PMC9695978 36432281

[29] Team RDC. R: a language and environment for statistical computing. Vienna, Austria: R Foundation for Statistical Computing, Vienna, Austria,; 2010.

[30] Bindea G, Mlecnik B, Hackl H, Charoentong P, Tosolini M, Kirilovsky A, et al. ClueGO: a cytoscape plug-in to decipher functionally grouped gene ontology and pathway annotation networks. Bioinformatics. 2009;25:1091–3. 19237447 10.1093/bioinformatics/btp101 PMC2666812

[31] Nam HY, Nam JH, Yoon G, Lee J-Y, Nam Y, Kang H-J et al. Ibrutinib suppresses LPS-induced neuroinflammatory responses in BV2 microglial cells and wild-type mice. J Neuroinflamm. 2018;15. 10.1186/s12974-018-1308-0 PMC6145206 30231870

[32] Giustarini D, Rossi R, Milzani A, Dalle-Donne I. Nitrite and nitrate measurement by Griess reagent in human plasma: evaluation of interferences and standardization. Methods Enzymol. 2008;440:361–380. Elsevier. 10.1016/S0076-6879(07)00823-3 18423230

[33] Chen T-W, Li H-P, Lee C-C, Gan R-C, Huang P-J, Wu TH, et al. ChIPseek, a web-based analysis tool for ChIP data. BMC Genomics. 2014;15:539. 24974934 10.1186/1471-2164-15-539 PMC4092222

[34] Qi X-M, Chen G. p38&gamma; MAPK inflammatory and metabolic signaling in physiology and disease. Cells. 2023;12:1674. 10.3390/cells12131674 PMC10341180 37443708

[35] Wang J, Liu Y, Gu Y, Liu C, Yang Y, Fan X et al. Function and inhibition of P38 MAP kinase signaling: targeting multiple inflammation diseases. Biochem Pharmacol. 2023:115973. 10.1016/j.bcp.2023.115973 38103797

[36] Wang G, Qian P, Jackson FR, Qian G, Wu G. Sequential activation of JAKs, STATs and xanthine dehydrogenase/oxidase by hypoxia in lung microvascular endothelial cells. Int J Biochem Cell Biol. 2008;40:461–70. 17920330 10.1016/j.biocel.2007.08.008 PMC2276459

[37] Osborn MJ, Rosen SM, Rothfield L, Zeleznick LD, Horecker BL. Lipopolysaccharide of the Gram-negative cell wall. Science. 1964;145:783–9. 14163315 10.1126/science.145.3634.783

[38] O’Neill LAJ, Golenbock D, Bowie AG. The history of toll-like receptors—redefining innate immunity. Nat Rev Immunol. 2013;13:453–60. 23681101 10.1038/nri3446

[39] Vaure Cl, Liu Y. A comparative review of toll-like receptor 4 expression and functionality in different animal species. Front Immunol. 2014; 5. 10.3389/fimmu.2014.00316 PMC4090903 25071777

[40] Lehnardt S, Massillon L, Follett P, Jensen FE, Ratan R, Rosenberg PA, et al. Activation of innate immunity in the CNS triggers neurodegeneration through a toll-like receptor 4-dependent pathway. PNAS. 2003;100:8514–9. 12824464 10.1073/pnas.1432609100 PMC166260

[41] Papageorgiou IE, Lewen A, Galow LV, Cesetti T, Scheffel J, Regen T, et al. TLR4-activated microglia require IFN-γ to induce severe neuronal dysfunction and death in situ. PNAS. 2016;113:212–7. 26699475 10.1073/pnas.1513853113 PMC4711883

[42] Sheppard O, Coleman MP, Durrant CS. Lipopolysaccharide-induced neuroinflammation induces presynaptic disruption through a direct action on brain tissue involving microglia-derived interleukin 1 beta. J Neuroinflamm. 2019;16:106. 10.1186/s12974-019-1490-8 PMC6525970 31103036

[43] Martiskainen H, Paldanius KMA, Natunen T, Takalo M, Marttinen M, Leskelä S, et al. DHCR24 exerts neuroprotection upon inflammation-induced neuronal death. J Neuroinflamm. 2017;14:215. 10.1186/s12974-017-0991-6 PMC5678793 29115990

[44] Guo X, Deng J, Zheng B, Liu H, Zhang Y, Ying Y et al. HDAC1 and HDAC2 regulate anti-inflammatory effects of anesthetic isoflurane in human monocytes. Immunol Cell Biol. 2020:imcb12318. 10.1111/imcb.12318 31950542

[45] Durham BS, Grigg R, Wood IC. Inhibition of histone deacetylase 1 or 2 reduces induced cytokine expression in microglia through a protein synthesis independent mechanism. J Neurochem. 2017;143:214–24. 28796285 10.1111/jnc.14144

[46] Cantley MD, Fairlie DP, Bartold PM, Marino V, Gupta PK, Haynes DR. Inhibiting histone deacetylase 1 suppresses both inflammation and bone loss in arthritis. Rheumatology. 2015;54:1713–23. 25832610 10.1093/rheumatology/kev022

[47] Kannan V, Brouwer N, Hanisch U-K, Regen T, Eggen BJL, Boddeke HWGM. Histone deacetylase inhibitors suppress immune activation in primary mouse microglia. J Neurosci Res. 2013;91:1133–42. 23686642 10.1002/jnr.23221

[48] Kadamb R, Mittal S, Bansal N, Batra H, Saluja D. Sin3: insight into its transcription regulatory functions. Eur J Cell Biol. 2013;92:237–46. 24189169 10.1016/j.ejcb.2013.09.001

[49] Oksuz O, Narendra V, Lee C-H, Descostes N, LeRoy G, Raviram R, et al. Capturing the onset of PRC2-mediated repressive domain formation. Mol Cell. 2018;70:1149–e11625. 29932905 10.1016/j.molcel.2018.05.023 PMC7700016

[50] Arthur JSC. MSK activation and physiological roles. Front Biosci. 2008;5866. 10.2741/3122 18508628

[51] Perdiguero E, Muñoz-Cánoves P. Transcriptional regulation by the p38 MAPK signaling pathway in mammalian cells. In: Posas F, Nebreda AR, editors. Stress-activated protein kinases. Volume 20. Berlin, Heidelberg: Springer Berlin Heidelberg; 2008. pp. 51–79.

[52] Cuenda A, Rousseau S. p38 MAP-Kinases pathway regulation, function and role in human diseases. Biochimica et biophysica acta (BBA) -. Mol Cell Res. 2007;1773:1358–75. 10.1016/j.bbamcr.2007.03.010 17481747

[53] Shapiro L, Dinarello CA. Osmotic regulation of cytokine synthesis in vitro. PNAS. 1995;92:12230–4. 8618875 10.1073/pnas.92.26.12230 PMC40330

[54] Beyaert R, Cuenda A, Vanden Berghe W, Plaisance S, Lee JC, Haegeman G, et al. The p38/RK mitogen-activated protein kinase pathway regulates interleukin-6 synthesis response to tumor necrosis factor. EMBO J. 1996;15:1914–23. 8617238 10.1002/j.1460-2075.1996.tb00542.x PMC450110

[55] Hwang D, Jang BC, Yu G, Boudreau M. Expression of mitogen-inducible cyclooxygenase induced by lipopolysaccharide. Biochem Pharmacol. 1997;54:87–96. 9296354 10.1016/S0006-2952(97)00154-8

[56] Kang YJ, Chen J, Otsuka M, Mols J, Ren S, Wang Y, et al. Macrophage deletion of p38α partially impairs lipopolysaccharide-induced cellular activation. J Immunol. 2008;180:5075–82. 18354233 10.4049/jimmunol.180.7.5075

[57] Pyo H, Jou I, Jung S, Hong S, Joe E-h. Mitogen-activated protein kinases activated by lipopolysaccharide and β-amyloid in cultured rat microglia. NeuroReport. 1998;9:871–4. 9579682 10.1097/00001756-199803300-00020

[58] Li Y, Liu L, Barger SW, Mrak RE, Griffin WST. Vitamin E suppression of microglial activation is neuroprotective. J Neurosci Res. 2001;66:163–70. 11592111 10.1002/jnr.1208 PMC3903400

[59] Combs CK, Karlo JC, Kao S-C, Landreth GE. β-Amyloid stimulation of microglia and monocytes results in TNFα-dependent expression of inducible nitric oxide synthase and neuronal apoptosis. J Neurosci. 2001;21:1179–88. 11160388 10.1523/JNEUROSCI.21-04-01179.2001 PMC6762255

[60] Hide I, Tanaka M, Inoue A, Nakajima K, Kohsaka S, Inoue K, et al. Extracellular ATP triggers tumor necrosis factor-α release from rat microglia. J Neurochem. 2000;75:965–72. 10936177 10.1046/j.1471-4159.2000.0750965.x

[61] Ryu J, Pyo H, Jou I, Joe E. Thrombin induces NO release from cultured rat microglia via protein kinase C, mitogen-activated protein kinase, and NF-κB. J Biol Chem. 2000;275:29955–9. 10893407 10.1074/jbc.M001220200

[62] Tikka T, Fiebich BL, Goldsteins G, Keinänen R, Koistinaho J. Minocycline, a tetracycline derivative, is neuroprotective against excitotoxicity by inhibiting activation and proliferation of microglia. J Neurosci. 2001;21:2580–8. 11306611 10.1523/JNEUROSCI.21-08-02580.2001 PMC6762519

[63] Chen Q, Guo J, Qiu T, Zhou J. Mechanism of ASK1 involvement in liver diseases and related potential therapeutic targets: a critical pathway molecule worth investigating. J Gastroenterol Hepatol. 2023;38:378–85. 36533997 10.1111/jgh.16087

[64] Ogier JM, Nayagam BA, Lockhart PJ. ASK1 inhibition: a therapeutic strategy with multi-system benefits. J Mol Med. 2020;98:335–48. 32060587 10.1007/s00109-020-01878-y PMC7080683

[65] Chen M, Qu X, Zhang Z, Wu H, Qin X, Li F, et al. Cross-talk between arg methylation and ser phosphorylation modulates apoptosis signal–regulating kinase 1 activation in endothelial cells. MBoC. 2016;27:1358–66. 26912789 10.1091/mbc.E15-10-0738 PMC4831888

[66] Hershko T, Korotayev K, Polager S, Ginsberg D. E2F1 modulates p38 MAPK phosphorylation via transcriptional regulation of ASK1 and Wip1. J Biol Chem. 2006;281:31309–16. 16912047 10.1074/jbc.M601758200

[67] Jiang C-F, Wen L-Z, Yin C, Xu W-P, Shi B, Zhang X et al. Apoptosis signal-regulating kinase 1 mediates the inhibitory effect of hepatocyte nuclear factor-4α on hepatocellular carcinoma. Oncotarget. 2016;7. 10.18632/oncotarget.8478 PMC5053659 27050273

[68] Maruyama T, Araki T, Kawarazaki Y, Naguro I, Heynen S, Aza-Blanc P, et al. Roquin-2 promotes ubiquitin-mediated degradation of ASK1 to regulate stress responses. Sci Signal. 2014;7:ra8–8. 24448648 10.1126/scisignal.2004822

[69] Nishida T, Hattori K, Watanabe K. The regulatory and signaling mechanisms of the ASK family. Adv Biol Regul. 2017;66:2–22. 28669716 10.1016/j.jbior.2017.05.004

[70] Tarapore RS, Yang Y, Katz JP. Restoring KLF5 in esophageal squamous cell cancer cells activates the JNK pathway leading to apoptosis and reduced cell survival. Neoplasia. 2013;15:472–IN3. 23633919 10.1593/neo.122126 PMC3638350

[71] Li W, Xiong Y, Shang C, Twu KY, Hang CT, Yang J, et al. Brg1 governs distinct pathways to direct multiple aspects of mammalian neural crest cell development. PNAS. 2013;110:1738–43. 23319608 10.1073/pnas.1218072110 PMC3562770

[72] Matsuzawa A, Saegusa K, Noguchi T, Sadamitsu C, Nishitoh H, Nagai S, et al. ROS-dependent activation of the TRAF6-ASK1-p38 pathway is selectively required for TLR4-mediated innate immunity. Nat Immunol. 2005;6:587–92. 15864310 10.1038/ni1200

[73] Into T, Shibata K-i. Apoptosis signal-regulating kinase 1‐mediated sustained p38 mitogen‐activated protein kinase activation regulates mycoplasmal lipoprotein‐and staphylococcal peptidoglycan‐triggered toll‐like receptor 2 signalling pathways. Cell Microbiol. 2005;7:1305–17. 16098218 10.1111/j.1462-5822.2005.00558.x

[74] Yuk J-M, Shin D-M, Yang C-S, Kim KH, An S-J, Rho J, et al. Role of apoptosis-regulating signal kinase 1 in innate immune responses by Mycobacterium bovis bacillus Calmette-Guérin. Immunol Cell Biol. 2009;87:100–7. 18852704 10.1038/icb.2008.74

[75] Yang C-S, Shin D-M, Lee H-M, Son JW, Lee SJ, Akira S, et al. ASK1-p38 MAPK-p47phox activation is essential for inflammatory responses during tuberculosis via TLR2-ROS signalling. Cell Microbiol. 2008;10:741–54. 18028450 10.1111/j.1462-5822.2007.01081.x

[76] Okazaki T. ASK family in infection and inflammatory disease. Adv Biol Regul. 2017;66:37–45. 29092784 10.1016/j.jbior.2017.10.001

[77] Guo X, Harada C, Namekata K, Matsuzawa A, Camps M, Ji H, et al. Regulation of the severity of neuroinflammation and demyelination by TLR-ASK1‐p38 pathway. EMBO Mol Med. 2010;2:504–15. 21064192 10.1002/emmm.201000103 PMC3377347

[78] Cheon SY, Kim EJ, Kim JM, Kam EH, Ko BW, Koo B-N. Regulation of microglia and macrophage polarization via apoptosis signal-regulating kinase 1 silencing after ischemic/hypoxic injury. Front Mol Neurosci. 2017;10:261. 28855861 10.3389/fnmol.2017.00261 PMC5557792

[79] Song J, Lee JE. ASK1 modulates the expression of microRNA Let7A in microglia under high glucose in vitro condition. Front Cell Neurosci. 2015;9. 10.3389/fncel.2015.00198 PMC4438231 26041997

[80] Lin H-Y, Tsai C-H, Lin C, Yeh W-L, Tsai C-F, Chang P-C, et al. Cobalt protoporphyrin upregulates cyclooxygenase-2 expression through a heme oxygenase-independent mechanism. Mol Neurobiol. 2016;53:4497–508. 26255181 10.1007/s12035-015-9376-y

[81] Shi X, Seldin David C, Garry Daniel J. Foxk1 recruits the Sds3 complex and represses gene expression in myogenic progenitors. Biochem J. 2012;446:349–57. 22716292 10.1042/BJ20120563 PMC4494662

