# Supplementary material for: SDS3 regulates microglial inflammation by modulating the expression of the upstream kinase ASK1 in the p38 MAPK signaling pathway
Source: Inflamm Res. 2024 Jul 15;73(9):1547–64. doi: 10.1007/s00011-024-01913-5 (PMC11349808; doi:10.1007/s00011-024-01913-5)
Supplement: Supplementary file 9 — Supplementary Material 9 [file 11_2024_1913_MOESM9_ESM.docx]

**Supplementary information**

**Journal name: Inflammation Research**

**Title:**

**SDS3 regulates microglial inflammation by modulating the expression of the upstream kinase ASK1 in the p38 MAPK signaling pathway.**

Jian Shen^1,#^, Wenjia Lai^3,#^, Zeyang Li^2^, Wenyuan Zhu^2^, Xue Bai^2^, Zihao Yang^2^, Qingsong Wang^2,*^ and Jianguo Ji^2,*^

1 Department of General Surgery, Beijing Chao-Yang Hospital, Capital Medical University, Beijing 100020, China

2 State Key Laboratory of Protein and Plant Gene Research, College of Life Sciences, Peking University, Beijing 100871, China

3 Division of Nanotechnology Development, National Center for Nanoscience and Technology, Beijing 100190, China

# These authors contributed equally to this work

Correspondence:

Qingsong Wang; State Key Laboratory of Protein and Plant Gene Research, College of Life Sciences, Peking University, Beijing 100871, China, Tel +86 10-62768978; E-mail wangqingsong@pku.edu.cn

Jianguo Ji; State Key Laboratory of Protein and Plant Gene Research, College of Life Sciences, Peking University, Beijing 100871, China, Tel +86 10-62755470; E-mail jijg@pku.edu.cn

**EXPERIMENTAL DETAILS**

**Real-time qPCR and Western blotting**

For qPCR analysis, after total RNA was extracted. A TransScript One-Step gDNA Removal and cDNA Synthesis SuperMix kit (Transgenbiotech, AT311) was used to synthesize cDNA. GoTaq qPCR master mix (Promega, A6002) and gene-specific primers were used for PCR amplification and fluorescence quantification. The relative mRNA levels of each gene were calculated using the (ΔΔCt) method based on the Ct values of each gene.

For Western blotting analysis, protein concentration was measured using a Pierce BCA Protein Assay kit (Thermo, Scientific 23225). Ten to 20 μg of protein was loaded onto 10–15% polyacrylamide gels and subjected to electrophoresis, followed by transfer onto a 0.2 µm PVDF membrane. Membranes were then blocked with 5% skim milk in PBST (1× PBS + 0.1% Tween 20) at room temperature for 1 hour. Primary antibodies were added and incubated overnight at 4°C (see Table S2 for antibody details). After washing with 0.1% PBST four times, secondary antibodies were added and incubated at room temperature for 1 hour. Membranes were washed four times with 0.1% PBST, and enhanced chemiluminescence Western blotting substrate (Millipore, WBKLS0500) was used to visualize protein bands. Band intensity was measured using ImageJ software, with β-Actin serving as the loading control. The relative levels of each protein were calculated by normalizing to the intensity of β-Actin expression and then comparing between samples.

**CRISPR–Cas9**

Using the Optimized CRISPR Design online tool (http://crispr.mit.edu), sgRNAs (single guide RNAs) were designed: ASK1 (CACCGATGGGCACCGAAGCCGGCGA, AAACTCGCCGGCTTCGGTGCCCATC) and control (CACCGGCGAGGTATTCGGCTCCGCG, AAACCGCGGAGCCGAATACCTCGCC). The synthesized oligonucleotides were annealed and ligated into the lentiCRISPR v2 plasmid, which was transformed into competent TransStbl3 Escherichia coli cells and cultured overnight on ampicillin LB agar plates. Plasmids were extracted and sequenced for verification.

The lentiCRISPR v2 plasmid containing sgRNA and the pMDLg/pRRE, pRSV-Rev, and pMD2.G plasmids were transfected into HEK 293T cells using polyethylenimine to package lentivirus. After 24–48 hours of transfection, the culture medium was collected by centrifugation to obtain the viral suspension, which was added to the BV2 cell culture medium. After 48 hours, puromycin (7 μg/mL) was added for selection. The medium was changed after 72 hours, and when cells reached 80–100% confluency, all cells were transferred to a 96-well plate for clonal selection.

Genomic DNA from the selected clonal cells was extracted using an EasyPure Genomic DNA kit (Transgenbiotech, EE101). The target genomic locus (ASK1) was amplified by PCR using Q5 High-Fidelity DNA Polymerase (NEB, M0491) and gene-specific primers (forward: GCGGCGGCCGCATGGCAGCTGCCTAGCCCG; reverse: GCGGGATCCTCGCCTCATTGATCACATAAGC). The PCR product was purified, digested, and ligated into the pBluescript II KS(-) plasmid, which was transformed into competent Trans5α E. coli cells and cultured overnight on ampicillin LB agar plates at 37°C. Six to ten colonies were selected and sequenced to verify the presence of the desired genomic locus.

**Nitric oxide measurement**

NaNO_2_ standard solutions were prepared at concentrations of 20, 10, 5, 2, 1, and 0 μM in the cell culture medium. In a 96-well plate, 50 μL of the standard solutions and the cell culture supernatant was added, followed by 50 μL of Griess Reagent I and 50 μL of Griess Reagent II. The mixture was vortexed, and the absorbance was measured at 540 nm using a microplate reader. A standard curve was plotted using the standard solution concentrations and the corresponding absorbance values, and the concentrations of each sample were calculated accordingly.

**Protein quantification by LC-MS/MS and data analysis**

TMT-labeled peptides were dissolved in 0.1% ammonia solution and subjected to high-pH, reversed-phase liquid chromatography using an UltiMate 3000 RSLCnano system (Thermo Scientific) and a Durashell C18 column (Agela). The mobile phase consisted of 0.1% ammonia (A) and 98% acetonitrile/0.1% ammonia (B), with a flow rate of 0.5 mL/min. The gradient was set as follows: 0–9% B for 6 min, 9–40% B for 90 min, and 40–80% B for 6 min. The eluate was collected in a deep 96-well plate with a volume of 0.5 mL per well. Fractions were pooled into 16 fractions, freeze-dried, and reconstituted with 0.2% formic acid.

Peptides were analyzed using an EASY-nLC 1200 liquid chromatography system (Thermo Scientific) coupled with an EASY-Spray C18 column (25 cm × 75 μM, 2-μm particle size) and an Orbitrap Fusion Lumos Tribrid mass spectrometer (Thermo Scientific) for liquid chromatography-tandem mass spectrometry analysis. The mobile phase for liquid chromatography consisted of 0.1% formic acid (A) and 80% acetonitrile/0.1% formic acid (B), with a flow rate of 300 nL/min. The following gradient was used: 0–6% B for 3 min, 6–9% B for 2 min, 9–32% B for 170 min, 32–50% B for 10 min, and 50–90% B for 1 min. The mass spectrometer was controlled using Thermo Xcalibur software, with MS1 scans performed at a resolution of 120,000 at 200 m/z in the scan range of 300–1,500 m/z. The automatic gain control (AGC) target was set to 1e6, and the maximum injection time was 100 ms. MS2 scans were performed using data-dependent acquisition with a top speed of 3 s/cycle. Higher-energy collisional dissociation fragmentation with 37% energy was used, and the resolution was set to 30,000 at 200 m/z. The AGC target was set to 1e5, and the maximum injection time was 100 ms. The fixed first mass was set to 100 m/z, and the isolation window was set to 1.2 m/z.

The liquid chromatography-tandem mass spectrometry results were analyzed using Proteome Discoverer 2.2 software, with the UniProt reference proteome UP000000589 (January 2019 release, one protein per gene, 22,287 entries) used as the database. The quantification mode was TMT6plex, allowing for a maximum of two missed cleavages. The fixed modifications were carbamidomethylation of cysteine residues and acetylation of protein N termini, whereas oxidation of methionine was considered a variable modification. The precursor ion mass tolerance and fragment ion mass tolerance were set to 10 ppm and 0.02 Da, respectively. Differential protein expression was determined using Perseus 1.6.2.3 software based on the total abundance values of each protein in different TMT channels.

**RNA-seq**

Total RNA was extracted, and RNA concentration and integrity were determined. Libraries were constructed using 1 μg of total RNA with an RNA integrity number greater than 7 and an NEBNext Ultra RNA Library Prep kit for Illumina. Paired-end sequencing (2 × 150 base pairs [bp]) was performed using a HiSeq 2500 system. Instrument control, image analysis, base calling, and format conversion were conducted using HiSeq Control and Off-Line Basecaller software. Data quality was assessed using FastQC software, and low-quality data were filtered using Cutadapt. Reads were then mapped to the reference genome using HiSat2 software, and transcript assembly was performed using StringTie.

**ChIP-seq and ChIP-qPCR**

For ChIP-seq analysis, after DNA quantification and quality assessment, 10 ng of DNA was used for library construction with an NEBNext Ultra II DNA Library Prep kit for Illumina. Libraries were sequenced using a HiSeq 2500 system with 2 × 150 bp paired-end reads. Instrument control, base calling, and format conversion were performed using HiSeq Control and Off-Line Basecaller software. Fastq format data were assessed for data quality using FastQC software, and low-quality data were filtered using Cutadapt. Reads were then mapped to the reference genome using bowtie2, and homer was used for peak scanning across the genome. Peak annotation was performed using the ChIPseek online tool (<http://chipseek.cgu.edu.tw>).

**SUPPLEMENTARY TABLES and FIRUGRES**

Table S1. qPCR primers.

Table S2. Antibody information.

Table S3. List of SDS3 target genes identified by ChIP-seq.

Table S4. Abundance ratios of all identified proteins (with unique peptides ≥2) from TMT-labeled quantitative proteomics.

Table S5. Ratios of 110 differentially expressed proteins identified by TMT-labeled quantitative proteomics.

Table S6. List of 13,195 genes with adjusted *p*-values identified by RNA-seq.

Table S7. Fold change values of differentially expressed genes identified by RNA-seq.

Table S8. List of downstream genes regulated by SDS3.

Fig. S1


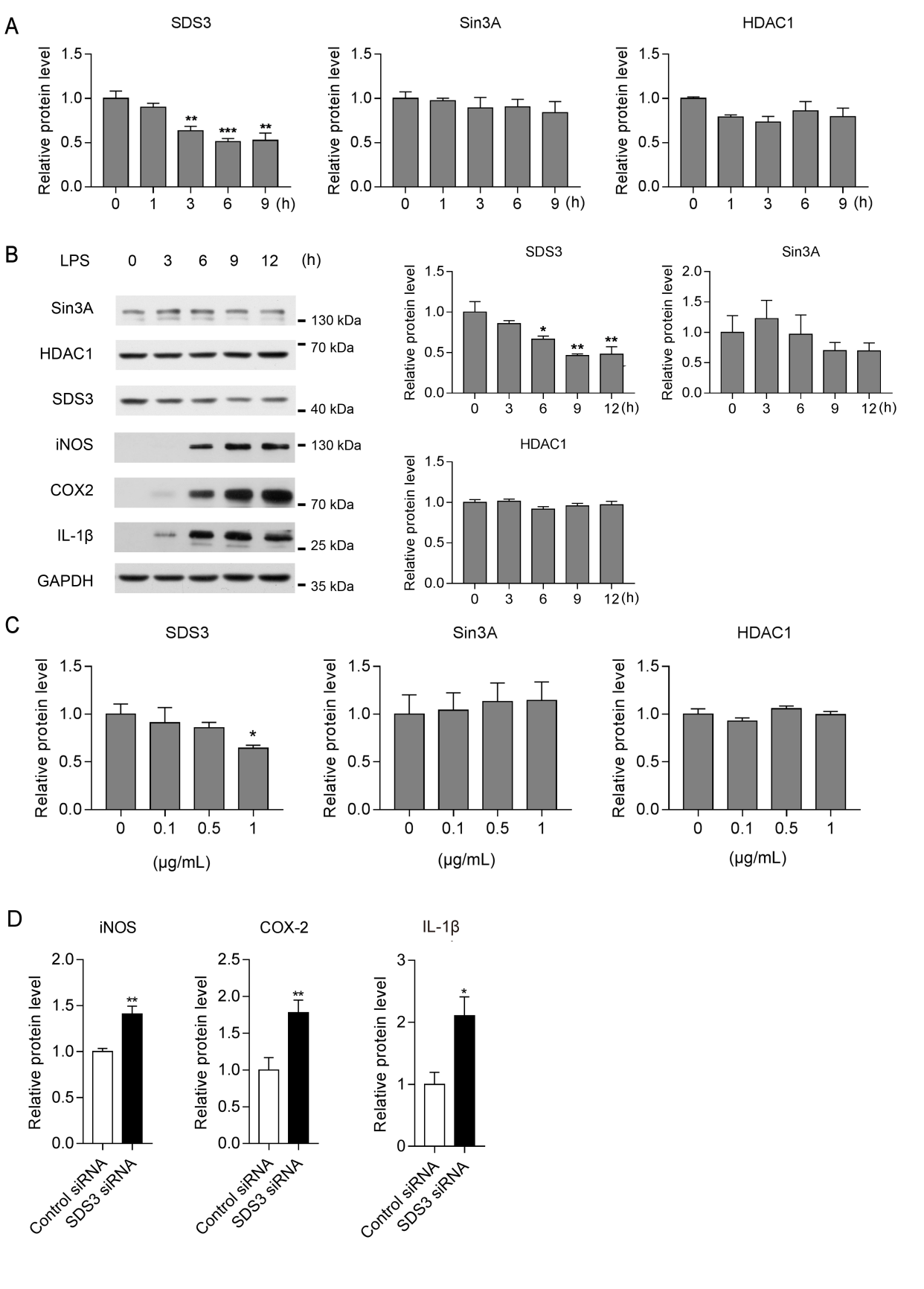


**Fig. S1.** Knockdown of *SDS3* enhances the LPS-induced microglial inflammatory response. (A) Expression of SDS3, Sin3A, and HDAC1 was detected using Western blotting after treating BV2 cells with 1 μg/mL LPS for different lengths of time. Data were analyzed by one-way analysis of variance, and β-Actin was used as the loading control. The control group represents 0 hours; *n* = 3; error bars represent mean ± standard error of the mean; ***p* < 0.01; ****p* < 0.001. (B) Expression of SDS3, Sin3A, HDAC1, iNOS, COX-2, and IL-1β was detected using Western blotting after treating primary mouse microglial cells with 1 μg/mL LPS for different lengths of time. Data were analyzed by one-way analysis of variance, and GAPDH was used as the loading control. The control group represents 0 hours; *n* = 3; error bars represent mean ± standard error of the mean; **p* < 0.05; ***p* < 0. 01. (C) SDS3, Sin3A, and HDAC1 expression was detected by Western blotting after treating BV2 cells with different concentrations of LPS for 6 hours. Data were analyzed by one-way analysis of variance, and β-Actin was used as the loading control. The control group represents 0 μg/mL; *n* = 3; error bars represent mean ± standard error of the mean; **p* < 0.05. (D) BV2 cells were transfected with SDS3 or Control siRNA, followed by treatment with 1 μg/mL LPS for 6 hours, and the expression of iNOS, COX-2, and IL-1β was detected by Western blotting. Data were analyzed using Student’s *t*-tests, and β-Actin was used as the loading control. The Control siRNA group represents the control group; *n* = 3; error bars represent mean ± standard error of the mean; **p* < 0.05; ***p* < 0.01.

Fig. S2


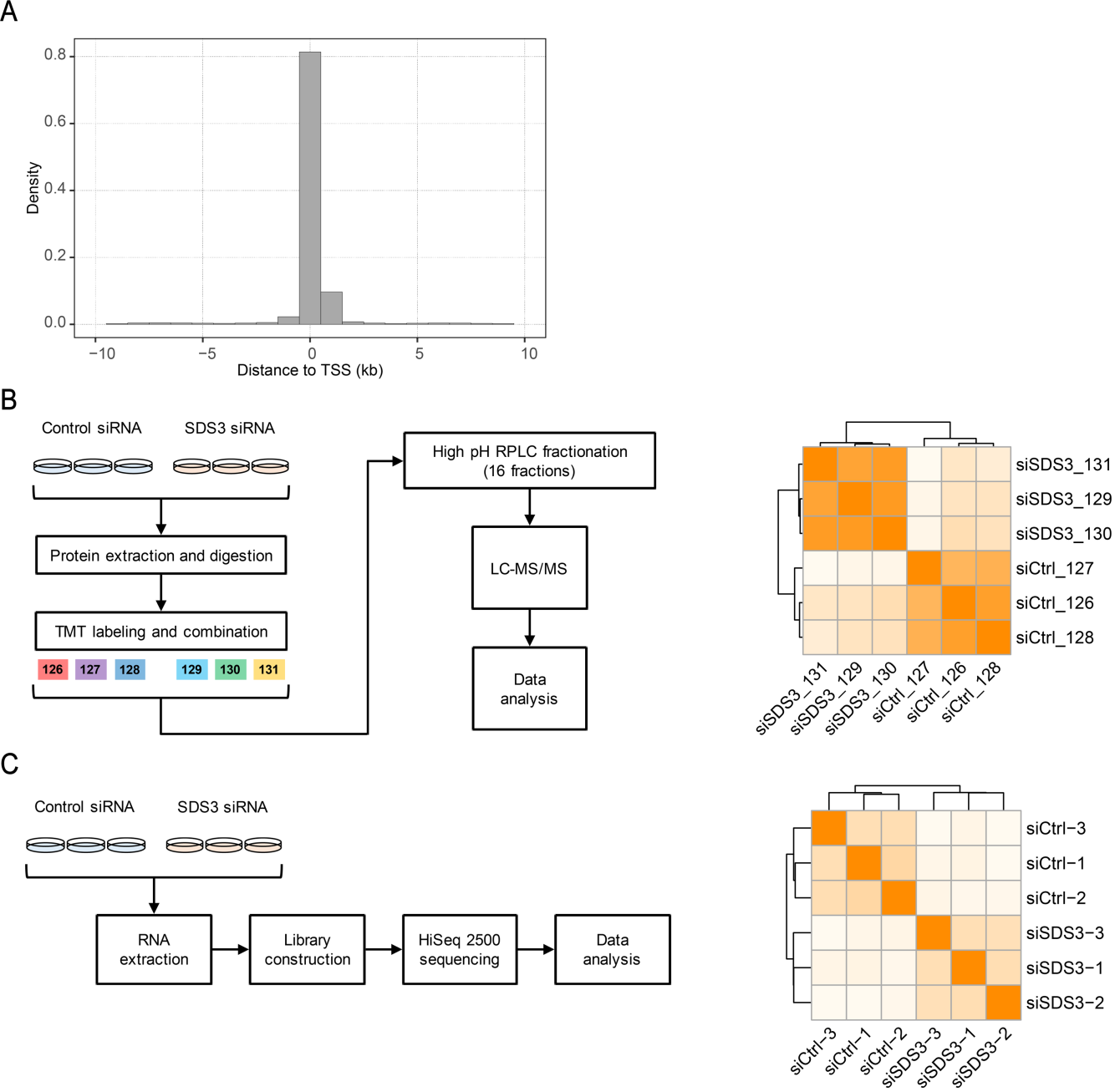


**Fig. S2.** Chromatin immunoprecipitation sequencing (ChIP-seq) and proteomic and transcriptomic analysis workflow. (A) Distribution of distances from SDS3 binding sites to the transcription start sites (TSSs) in the genome. ChIP-seq analysis was performed in BV2 cells to detect SDS3 binding sites, and the distances of these regions to the TSS were calculated. Distribution is represented as a density plot with a bin size of 1 kb. (B) Proteomic analysis workflow in *SDS3*-knockdown cells. BV2 cells were transfected with Control or SDS3 siRNA, followed by protein extraction and enzymatic digestion into peptides. Samples from each group were labeled with 6-plex tandem mass tags (TMT) reagents, and the labeled peptides were mixed. The mixed peptide samples were subjected to high-pH, reversed-phase liquid chromatography (RPLC) to separate into 16 fractions, followed by liquid chromatography-tandem mass spectrometry (LC-MS/MS) analysis. Pearson correlation coefficients were calculated based on the log_2_ abundance of each protein quantified by TMT labeling, and hierarchical clustering analysis was performed (right). The results showed good correlation among biological replicates and effective discrimination between experimental and control samples, indicating good reproducibility of the proteomic data. (C) Transcriptomic analysis workflow in *SDS3*-knockdown cells. BV2 cells were transfected with Control or SDS3 siRNA, followed by total RNA extraction and library construction. Libraries were sequenced using the HiSeq 2500 sequencing system, and the sequencing results were subjected to subsequent data analysis (left). Fragments per kilobase per million (FPKM) values were calculated based on the read count of each gene obtained from sequencing, followed by log_2_ transformation. Pearson correlation coefficients were calculated based on the log_2_ FPKM values of each gene among all samples, and hierarchical clustering analysis was performed (right). The results demonstrated good correlation among biological replicates and effective discrimination between experimental and control samples, indicating good reproducibility of the transcriptomic data.

Fig. S3


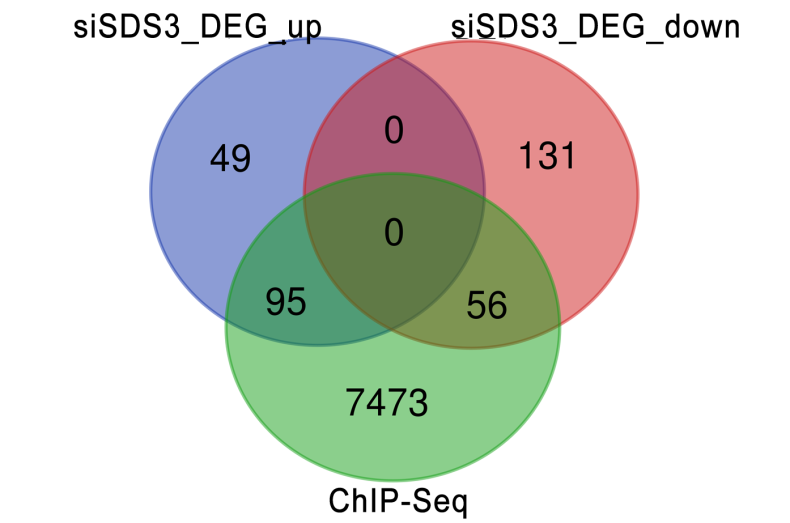


**Fig. S3.** Venn plot illustrating the overlap of genes identified from ChIP-seq analysis and differentially expressed genes (siSDS3_DEG) from transcriptomic analysis. siSDS3_DEG_up represents upregulated genes, and siSDS3_DEG_down represents downregulated genes.

Fig. S4


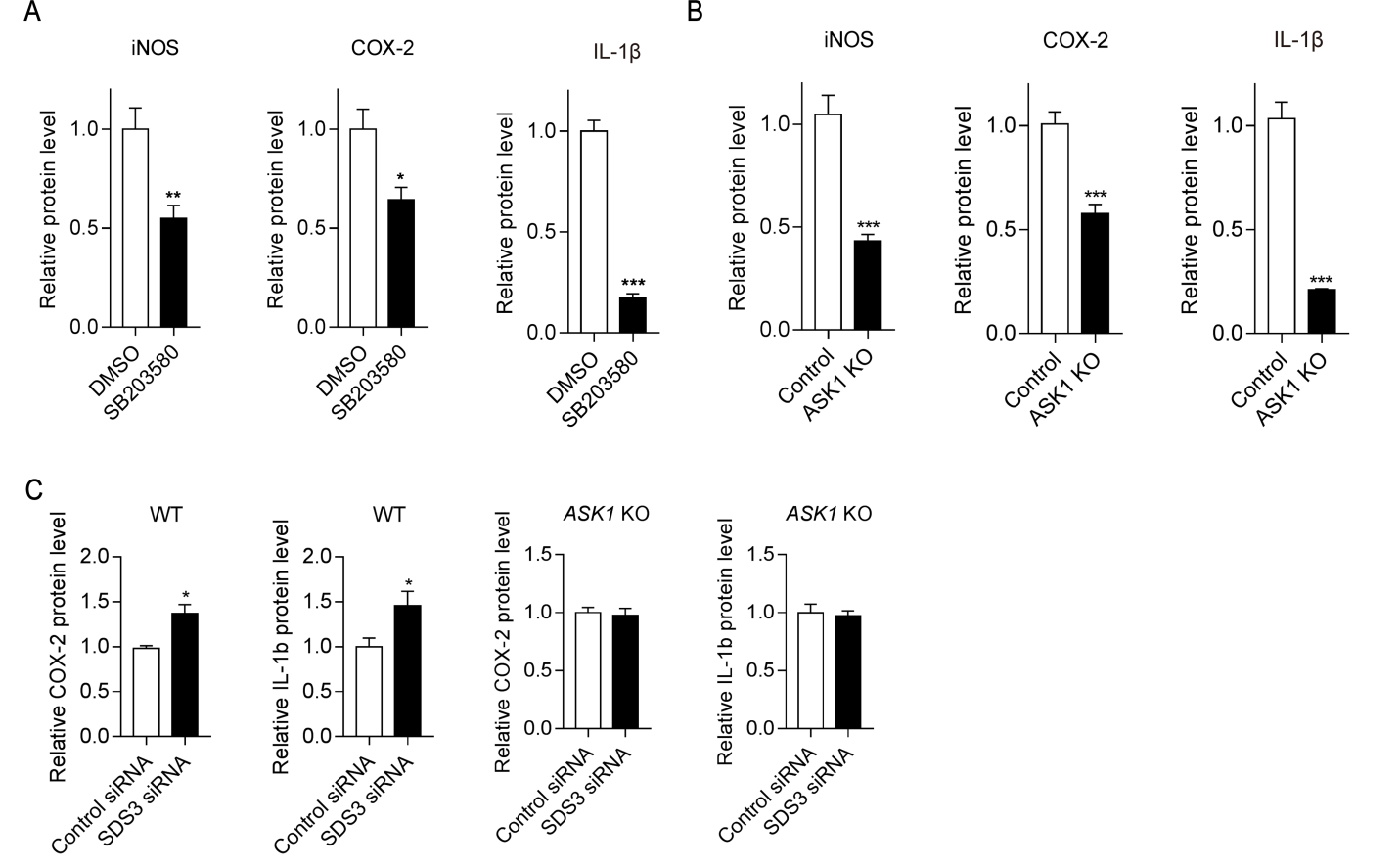


**Fig. S4.** Statistical analysis of relative protein levels of inflammatory factors as determined by Western blotting. (A) BV2 cells were treated with 20 μM SB203580 or DMSO for 1 hour, followed by treatment with 1 μg/mL LPS for 6 hours. Data were analyzed by Student’s *t*-tests, and β-Actin was used as the loading control. The DMSO group represents the control group; *n* = 3; error bars represent mean ± SEM; ***p* < 0.01; ****p* < 0.001. (B) Expression of iNOS, COX-2, and IL-1β following treatment of *ASK1*-KO and WT BV2 cells with 1 μg/mL LPS for 6 hours. β-Actin was used as the internal reference protein. WT BV2 cells treated with LPS represents the control group. Data were analyzed by Student’s *t*-tests; *n* = 3; error bars represent mean ± SEM. ****P* < 0.001. (C) *ASK1*-KO and WT BV2 cells were transfected with SDS3 or Control siRNA, followed by treatment with 1 μg/mL LPS for 6 hours. Data were analyzed by Student’s *t*-tests. Separate statistical analyses were performed for *ASK1*-KO and WT BV2 cells due to different exposure times. β-Actin was used as the loading control. WT BV2 transfected with Control siRNA represents the control group; *n* = 3; error bars represent mean ± SEM. **P* < 0.05.
